# Divergent maturational patterns of the infant bacterial and fungal gut microbiome in the first year of life are associated with inter-kingdom community dynamics and infant nutrition

**DOI:** 10.1186/s40168-023-01735-3

**Published:** 2024-02-07

**Authors:** Emily M. Mercer, Hena R. Ramay, Shirin Moossavi, Isabelle Laforest-Lapointe, Myrtha E. Reyna, Allan B. Becker, Elinor Simons, Piush J. Mandhane, Stuart E. Turvey, Theo J. Moraes, Malcolm R. Sears, Padmaja Subbarao, Meghan B. Azad, Marie-Claire Arrieta

**Affiliations:** 1 Department of Physiology and Pharmacology, University of Calgary, Calgary, AB Canada; 2 Department of Pediatrics, University of Calgary, Calgary, AB Canada; 3 International Microbiome Center, University of Calgary, Calgary, AB Canada; 4 Snyder Institute for Chronic Diseases, University of Calgary, Calgary, AB Canada; 5 Alberta Children’s Hospital Research Institute (ACHRI), Calgary, AB Canada; 6 Department of Microbiology, Immunology and Transplantation, Rega Institute, KU Leuven, Louvain, Belgium; 7 VIB Center for Microbiology, VIB, Louvain, Belgium; 8 Department of Biology, University of Sherbrooke, Sherbrooke, Québec Canada; 9 Dalla Lana School of Public Health, University of Toronto, Toronto, ON Canada; 10 Department of Translational Medicine, Hospital for Sick Children, Toronto, ON Canada; 11 Department of Pediatrics and Child Health, University of Manitoba, Winnipeg, MB Canada; 12 Children’s Hospital Research Institute of Manitoba, Winnipeg, MB Canada; 13 Department of Pediatrics, University of Alberta, Edmonton, AB Canada; 14 Department of Pediatrics, BC Children’s Hospital, University of British Columbia, Vancouver, BC Canada; 15 Department of Medicine, McMaster University, Hamilton, ON Canada; 16 Manitoba Interdisciplinary Lactation Centre (MILC), Winnipeg, MB Canada

**Keywords:** Gut microbiome, Gut mycobiome, Alpha diversity, Early life, Microbial succession, Microbiome maturation, Colonization patterns, Inter-kingdom dynamics, Gut fungi

## Abstract

**Background:**

The gut microbiome undergoes primary ecological succession over the course of early life before achieving ecosystem stability around 3 years of age. These maturational patterns have been well-characterized for bacteria, but limited descriptions exist for other microbiota members, such as fungi. Further, our current understanding of the prevalence of different patterns of bacterial and fungal microbiome maturation and how inter-kingdom dynamics influence early-life microbiome establishment is limited.

**Results:**

We examined individual shifts in bacterial and fungal alpha diversity from 3 to 12 months of age in 100 infants from the CHILD Cohort Study. We identified divergent patterns of gut bacterial or fungal microbiome maturation in over 40% of infants, which were characterized by differences in community composition, inter-kingdom dynamics, and microbe-derived metabolites in urine, suggestive of alterations in the timing of ecosystem transitions. Known microbiome-modifying factors, such as formula feeding and delivery by C-section, were associated with atypical bacterial, but not fungal, microbiome maturation patterns. Instead, fungal microbiome maturation was influenced by prenatal exposure to artificially sweetened beverages and the bacterial microbiome, emphasizing the importance of inter-kingdom dynamics in early-life colonization patterns.

**Conclusions:**

These findings highlight the ecological and environmental factors underlying atypical patterns of microbiome maturation in infants, and the need to incorporate multi-kingdom and individual-level perspectives in microbiome research to improve our understandings of gut microbiome maturation patterns in early life and how they relate to host health.

Video Abstract

**Supplementary Information:**

The online version contains supplementary material available at 10.1186/s40168-023-01735-3.

## Background

In early life, the gut microbiome undergoes successional shifts in composition leading to the establishment of stable microbial communities around 3 years of age [1]. This begins as a primary succession event where pioneer microbes colonize the sterile gut upon birth, and subsequently, increase in taxonomic and/or functional diversity over time, depending on the microbial kingdom in question [2–4]. For bacteria, these successional events are shaped by host-driven (intestinal pH, oxygen pressure, glycan expression in breastmilk and on the gut mucosa (*FUT2* secretor status), etc.) and environmental factors, such as mode of birth, breastfeeding, introduction of solid foods, and antibiotic use [5–8]. Meanwhile, few characterizations of the role of these factors in gut fungal succession have been performed, but current research suggests the mycobiome may be more strongly influenced by dietary and geographical factors [3, 9]. Like other microbial ecosystems, the mammalian intestine is a stage for inter-kingdom interactions, in which gut fungi play important ecological roles in shaping bacterial microbiomes and vice versa [10–15]. However, our knowledge of how bacterial and fungal interactions influence successional shifts in diversity during early-life microbiome establishment remains very limited.

Compositionally, the patterns of bacterial microbiome maturation have been well-described, and include increases in alpha (within-individual) diversity and decreases in beta (between-individual) diversity, indicating reductions in the inter-individual variability of microbiome composition with age [16–20]. This is further characterized by transitions from communities abundant in *Enterobacteriaceae* and *Bifidobacteriaceae* in the first months of life, towards those comprised of *Bacteroidaceae*, *Lachnospiraceae*, and *Ruminococcaceae* over the first 2–3 years [16–20]. For fungi, descriptions of mycobiome maturation remain limited and confounded by small sample sizes, with reports showing variable (increasing, decreasing, or stable) changes in alpha diversity and increases in beta diversity in early-life [3, 9, 21–28]. This indicates greater inter-individual variability is observed in mycobiome composition with age, in contrast to the patterns observed for bacteria. Despite this, consistencies in early and late colonizers have been reported during primary succession. This includes early colonization with fungi such as *Candida*, *Malassezia*, *Cladosporium*, and *Debaryomyces*, followed by a shift towards communities dominated by *Saccharomyces* with the introduction of solid foods into the infant diet [3, 9, 21–28].

Deviations from typical patterns of bacterial microbiome maturation during the first year of life have been reported in association with disease states, such as type 1 diabetes, asthma, and celiac disease [17, 29, 30], highlighting the need to better understand dysbiotic maturational trajectories. Early life is regarded as a critical window when microbial colonization exerts potent influences on human development [31, 32]. In the absence of eubiotic patterns of microbiome establishment, the developmental programming of host physiology may be altered, potentially having detrimental and lasting implications on host health [31, 32]. When examined through this lens, it is critical to both characterize and be able to distinguish the continuum of typical vs. atypical gut microbiome maturation patterns in early life. Beyond disease paradigms, our understanding of the variability in patterns of gut microbial colonization across infants is further limited by a focus on group-based analyses in microbiome research. This includes exploring microbiome changes across infants based on specific factors (e.g., delivery mode, nutrition, antibiotic exposure) or between different cohorts (e.g., geographically), but typically does not include examining how these factors influence the microbiome within each individual. While this streamlines the handling of large microbiome datasets, it inherently limits our understanding of individual differences in patterns of microbiome maturation. In parallel, research efforts have primarily focused on the bacterial microbiome, despite the co-existence of several other microbial kingdoms contributing to the makeup of the gut microbiome [33]. Together, the shortage of individual-level and multi-kingdom perspectives in microbiome research to date have limited our understanding of eubiotic vs. dysbiotic patterns of gut microbiome maturation in early life.

In this work, we begin to address this knowledge gap by evaluating both bacterial and fungal gut microbiome maturation patterns in 100 infants from the CHILD Cohort Study [34] over the first year of life using individual-level and multi-kingdom perspectives. Using a similar approach for mycobiome maturation, we have previously shown increasing vs. decreasing fungal richness over the first year of life is differentially associated with early childhood body mass index (BMI) z-scores in this sub-cohort, with this relationship being mediated by the influence of antibiotics exposure, maternal BMI and diet, and bacterial beta diversity [35]. Here, we determined that divergent patterns of bacterial and fungal gut microbiome maturation are more common in term-born infants than previously considered, occurring in over 40% of infants in this sub-cohort, which are characterized by differences in ecological and metabolic properties that may reflect altered rates of microbiome maturation. These maturational trajectories were differentially associated with prenatal, environmental, genetic, and ecological factors, highlighting the need to consider the variable influences these factors have on bacterial vs. fungal gut microbiome maturation in early life.

## Materials & methods

### Study design & population

We investigated the early-life microbiome in 100 infants from the CHILD Cohort Study – a prospective population-based birth cohort recruiting women with healthy singleton pregnancies who delivered after 35 weeks’ gestation (*n* = 3,264) [34]. Mother-infant dyads were recruited from 2008 to 2012 across four Canadian provinces and study sites: Vancouver (British Columbia), Edmonton (Alberta), Toronto (Ontario), and Winnipeg (Manitoba) and two adjacent rural towns, Morden and Winkler [34]. In this study, we evaluated a sub-cohort of 100 mother-infant dyads previously selected for a nested case–control study on the influence of maternal artificially sweetened beverage consumption during gestation on the infant bacterial gut microbiome [36]. Mother-infant dyads were divided equally between mothers who reported little to no artificially sweetened beverage consumption (less than one per month) or high artificially sweetened beverage consumption (one or more per day) during pregnancy. These groups were balanced for six potentially confounding factors known to influence the gut microbiome in early life: infant sex assigned at birth, delivery mode, breastfeeding status at 3 and 12 months, infant antibiotic exposure before 12 months (exposure prior to 3 months was an exclusion criterion), and maternal BMI [36]. The study was approved by the University of Calgary Conjoint Health Research Ethics Board and ethics committees at the Hospital for Sick Children, and the Universities of Manitoba, Alberta, and British Columbia. Written informed consent was obtained from mothers during study enrollment and prior to data collection at each subsequent visit.

### Infant, early-life & maternal factors

We considered the influence of infant, early-life, and maternal factors with known influences on the gut microbiome, while controlling for maternal diet and artificially sweetened beverage consumption during gestation given the original selection criteria of this sub-cohort [36]. Infant sex assigned at birth, gestational age, delivery mode, and prenatal, intrapartum, and early life (0–12 months) antibiotics exposure were recorded from infant and maternal medical records. Infant feeding was reported using a standardized questionnaire at 3, 6, and 12 months [34]. This included breastfeeding status at 3 months, breastfeeding duration or age at breastfeeding cessation (months), and age at introduction of solid foods (months). Breastfeeding status at 3 months was classified as ‘‘exclusive’’ (human milk only), “partial” (human milk supplemented with formula milk or solid foods), or ‘‘none’’ (no human milk). Infant and maternal secretor status was determined from the single nucleotide polymorphism (SNP) rs601338 in the *FUT2* gene and classified based on genotype: “AA” (homozygous non-secretor), “AG” (heterozygous secretor), and “GG” (homozygous secretor). Due to a limited racial distribution in this sub-cohort (*n* = 81 Caucasian vs. *n* = 10 Asian mothers), we were not powered to examine the rs1047781 SNP associated with secretor status in Asian populations, but only one mother had the missense genotype for this locus [37]. Maternal diet was evaluated using a validated food frequency questionnaire in the second or third trimester of pregnancy, with modifications to capture typical dietary patterns throughout the current pregnancy [34, 38]. The Healthy Eating Index (HEI) was derived from the food frequency questionnaire based on the 2010 guidelines [39] and used as a measure of maternal diet quality. Artificially sweetened beverage consumption during pregnancy was determined based on consumption of diet sodas (1 serving = 355 mL or one can) or artificial sweetener added to tea or coffee (1 serving = 1 packet) [36]. Maternal BMI was determined using measured height and self-reported pre-pregnancy weight. Infant BMI z-scores were derived from weight and length measurements at 3 and 12 months recorded by CHILD Cohort Study staff based on the 2011 World Health Organization (WHO) standards [40]. Participant characteristics have been summarized in Table 1.

### Sample collection & processing

Infant fecal and urine samples (*n* = 200 each) were collected from soiled diapers by CHILD Cohort Study staff at the 3-month home visit and 12-month clinic visit for each participant, using standardized methods across both timepoints [41]. For home visits, samples were transported on ice back to the laboratory and frozen within 8 h of collection. All samples were stored at -80 °C until further processing [41].

### Fecal DNA extractions

Genomic DNA was extracted from fecal samples using the DNeasy PowerSoil Pro Kit (Qiagen, Germany) according to the manufacturer’s instructions. Extraction kit negatives were processed alongside fecal samples and extractions for all fecal samples (*n* = 200) were performed during the same period. DNA concentrations and quality were quantified using a NanoDrop Lite spectrophotometer (Thermo Scientfic, USA). DNA was stored at -20 °C until further processing.

### 16S & ITS2 rRNA gene sequencing

16S and ITS2 rRNA gene sequencing of fecal DNA was performed by Microbiome Insights (Vancouver, Canada). PCR amplification of the V4 region of the bacterial 16S rRNA gene with 515F/806R primers [42] and the fungal internal transcribed spacer 2 (ITS2) region with ITS1F/ITS4 primers [43] was performed using Phusion Hot Start II DNA Polymerase (Thermo Scientific, USA) to generate ready-to-pool, dual-indexed amplicon libraries, as previously described [44]. Microbial contamination was controlled for throughout the PCR and downstream sequencing steps using mock communities with defined amounts of select bacteria or fungi and controls lacking microbial DNA. The pooled and indexed amplicon libraries were denatured, diluted, and sequenced in a single run on an Illumina MiSeq (Illumina Inc., USA) in paired-end modus.

Sequence processing was performed in R v.4.2.1 [45] using the *DADA2* v.1.26.0 pipelines for 16S and ITS2 data [46]. The median read count of samples after *DADA2* processing was 33,103 (27,700–37,369) for 16S and 15,740 (9,625–29,954) for ITS2 (Table S1 and Figure S1A-B). Taxonomic assignment based on amplicon sequence variants (ASVs) was performed using the following databases at 99% sequence similarity: SILVA v.132 [47] for bacteria (16S) and UNITE v.8.0 [48] for fungi (ITS2). Pre-processing of bacterial and fungal data was performed using *phyloseq* v.1.42.0 [49] and has been previously reported [35, 36]. In brief, 954 unique bacterial ASVs were identified. Samples were filtered to remove those with less than 1,000 reads, singletons, and ASVs appearing less than 2 times in a minimum of 10% of the samples, leaving 540 bacterial ASVs for downstream analysis [36]. For fungi, 3,328 unique ASVs were identified. The same filtering criteria were applied with the following modifications: ASVs belonging to the kingdom Plantae were removed and a higher threshold of less than 2,000 reads was applied based on the lower sequencing depth of these samples (Figure S1C-D). 604 unique fungal ASVs remained in the dataset for downstream analyses [35].

### Untargeted urine metabolomics

Untargeted quantitative metabolomics of urine samples was performed at the Calgary Metabolomics Research Facility (Calgary, Canada), using a combination of direct injection mass spectrometry (MS) with a reverse-phase liquid chromatography (LC)-MS/MS assay, as described previously [36]. This assay enables the identification and quantification of up to 150 metabolites, including sugars, amino acids, acylcarnitines, biogenic amines and derivatives, uremic toxins, glycerophospholipids, and sphingolipids [50, 51]. Isotope-labeled internal standards and quality control standards were used for metabolite quantification. Mass spectrometry was performed on a 4000 QTRAP® LC–MS/MS mass spectrometer (SCIEX, USA) equipped with a 1260 Infinity II LC System (Agilent Technologies, USA) using a sequential combination of LC and direct injection approaches.

### Exclusion of data

Infants lacking samples at both timepoints were excluded from all downstream analyses involving bacterial (*n* = 2) and/or fungal (*n* = 9) alpha diversity trends, given two samples were required to determine trend directionality. Infants with an atypical alpha diversity trend for both bacteria and fungi (*n* = 2) were excluded from inter-kingdom co-occurrence network analyses due to sample size limitations. Further, samples with missing data for infant, maternal, early-life, or ecological covariates were excluded from random forest and logistic regression analyses. This included prenatal antibiotics (*n* = 2), intrapartum antibiotics (*n* = 3), and introduction of solid foods (*n* = 2) for both bacteria and fungi analyses; fungal alpha (Shannon) and beta (PCoA1) diversity at 3 (*n* = 5) and 12 (*n* = 5) months for the bacterial alpha diversity trend random forest; and bacterial alpha (Shannon) and beta (PCoA1) diversity at 3 (*n* = 1) and 12 months (*n* = 1) for the fungal alpha diversity random forest.

### Statistical analysis

Bacterial and fungal alpha diversity were quantified at 3 and 12 months using the Shannon (diversity) and Chao1 (richness) indices with *phyloseq* v.1.42.0 [49] and reported as mean and standard deviation (SD). Changes in alpha diversity with age were assessed by Mann–Whitney U test, after determining data was non-normally distributed using the Shapiro–Wilk test. To assess individual-level shifts in alpha diversity, Shannon and Chao1 metrics were classified into “increase”, “decrease”, or “unchanged” categories based on the change in these metrics per infant from 3 to 12 months and assessed by paired t-test. Bacterial and fungal beta diversity were evaluated using the Bray–Curtis dissimilarity index with variance-stabilizing transformation and differences based on age and alpha diversity trend were assessed by permutational analysis of variance (PERMANOVA) using *vegan* v.2.6.4 [52]. Multivariate homogeneity of groups dispersions (beta dispersion) was also assessed by age and alpha diversity trend using a permutation test with *vegan* v.2.6.4 [52].

The relative abundance of the 15 most abundant bacterial and fungal genera were compared by age and alpha diversity trend. Relative abundances were center log-ratio (CLR) transformed and zeros were handled by adding a small pseudo-count using *microbiome* v.1.22 [53] to control for compositionality prior to assessing statistical differences in abundance. Normality was determined using the Shapiro–Wilk test. Non-normally distributed CLR-transformed abundances were assessed by Mann–Whitney U test and normally distributed abundances were assessed for equality of variance using the F-test, then evaluated by Student’s t-test or Welch’s Two Sample t-test if the variance was equal or unequal, respectively. Differential abundance analysis by age and alpha diversity trend was performed at the ASV level for bacteria and fungi using *DESeq2* v.1.38.3 [54]. Bacterial and fungal count datasets underwent variance-stabilizing transformation and were filtered for taxa with at least 5,000 reads summed across all samples to limit the overrepresentation of rare ASVs. The typical bacterial or fungal alpha diversity trend was set as the reference level to identify ASVs that were differentially abundant in infants with an atypical alpha diversity trend at 3 and 12 months.

Random forest was performed to identify factors predictive of bacterial and fungal alpha diversity trend direction using 10-fold cross-validation, 500 trees, and 1,000 permutations by the *randomForest* v.4.6.14 and *caret* v.6.0.90 packages [55, 56]. Factors known to be associated with microbiome maturation in early life were included [19, 20, 57], alongside bacterial or fungal alpha and beta diversity measures to evaluate for inter-kingdom influences. The mean decreasing Gini index (GI) was computed to identify factors most strongly associated with bacterial and fungal alpha diversity trends. Next, multivariable logistic regression examining factors associated with an atypical bacterial (decreasing) or fungal (increasing) alpha diversity trend was performed using *stats* v.4.1.1 [58] to determine the directionality of associations between alpha diversity trends and early-life factors. Logistic regression models were assessed for multi-collinearity and optimized using *performance* v.0.8.0 [59]. Bacterial and fungal alpha and beta diversity measures were excluded from this analysis to prevent overfitting and enable focused investigations of how clinical and early-life factors influence changes in alpha diversity over the first year of life. The results are presented for each factor as the log-transformed odds ratio (OR) and 95% confidence interval (CI). The typical bacterial (increasing) and fungal (decreasing) alpha diversity trend was set as the reference level for both random forest and logistic regression analyses. Any potential confounding effects based on the sub-cohort selection criteria were controlled for by including maternal dietary factors (HEI and gestational artificially sweetened beverage consumption) in these analyses.

Urine metabolites were evaluated by age and alpha diversity trend using the web-based server, MetaboAnalyst [60]. Metabolite concentrations were normalized using the median, log-transformed, and pareto scaled (mean-centered and divided by the square root of the standard deviation of each metabolite). Differences in normalized urine metabolite concentrations between increasing and decreasing bacterial and fungal alpha diversity trends at 3 and 12 months were assessed by t-test using a false discovery rate (FDR) threshold of < 0.1.

Bacterial and fungal inter-kingdom co-occurrence network analysis was performed at the species level using *NetCoMi* v.1.1.0 [61]. Networks were generated based on the combination of bacterial and fungal alpha diversity trends exhibited by each infant and were allocated into the following groups: typical (increasing bacterial and decreasing fungal alpha diversity; *n* = 50), bacteria atypical (decreasing bacterial and fungal alpha diversity; *n* = 21), fungi atypical (increasing bacterial and fungal alpha diversity; *n* = 16), or both atypical (decreasing bacterial and increasing fungal alpha diversity; *n* = 2). Infants who displayed atypical alpha diversity trends for both bacteria and fungi were excluded from the main analysis due to sample size limitations, but were included when comparing typical vs. all atypical patterns of alpha diversity changes in combination. Networks were constructed using variance-stabilizing transformed count data, pseudo zero handling, and a Pearson correlation threshold of ± 0.4, then were assessed using a fast greedy clustering algorithm. Node size was determined based on degree centrality. Hub taxa were identified as those having the highest betweenness centrality. Pair-wise network comparisons were made between typical (inverse), bacteria atypical, fungi atypical, and all atypical overall alpha diversity patterns combined at 3 and 12 months by calculating the following measures using 5,000 permutations: centrality (degree, betweenness, closeness, eigenvector), clustering coefficient, modularity, edge density, positive edge percentage, connectivity (natural, vertex, edge), average dissimilarity, average path length, and hub taxa. Co-occurrence networks at 3 and 12 months were also generated for bacteria and fungi in isolation using the same approach to examine co-occurrence dynamics amongst microbiome members of the same kingdom between increasing and decreasing alpha diversity trends.

## Results

### Participant characteristics

This work was performed as a secondary analysis of a previously published nested case–control study examining the influence of prenatal consumption of artificial sweeteners on infant bacterial microbiome maturation patterns and body mass index (BMI) in early life. Of the 100 infants evaluated in this study, 46% were female and 64% were delivered vaginally. Antibiotic exposure in the prenatal and postnatal periods occurred in 12% and 20% of mother-infant dyads, respectively, while 52% were exposed to intrapartum antibiotics. Breastfeeding status at 3 months was approximately equally distributed between none (36%), partial (30%), and exclusive (34%), with a mean breastfeeding duration of 7.8 ± 7.3 months. Introduction of solid foods into the infant diet occurred at 4.8 ± 1.2 months. The proportion of mothers and infants who secrete ABO histo-blood group antigens in other bodily fluids, such as breastmilk and the intestinal mucosa, was determined by *FUT2* genotype [62]. Approximately 80% of mothers and infants were secretors, with secretor status balanced by genotype (AA or non-secretor: 24% of infants and 22% of mothers; AG or heterozygous secretor: 39% of infants and 41% of mothers; GG or homozygous secretor: 37% of infants and mothers; Table 1), consistent with the genotypic distribution of *FUT2* secretor status in the general population [63].

**Table 1:** Characteristics of mother-infant dyads from the CHILD cohort included in this analysis (*n* = 100)

**Characteristics**	**n (%) or Mean ± SD**
Infant Sex (Female)	46 (46%)
Mode of Birth (Vaginal)	64 (64%)
Prenatal Antibiotics^a^	12 (12%)
Intrapartum Antibiotics^b^	50 (52%)
Infant Antibiotic Exposure (3–12 months)	20 (20%)
Breastfeeding Status at 3 Months	
None	36 (36%)
Partial	30 (30%)
Exclusive	34 (34%)
Breastfeeding Duration (months)	7.8 ± 7.3
Age at Introduction of Solid Foods (months)^a^	4.8 ± 1.2
Infant *FUT2* Genotype^c^	
AA	24 (24%)
AG	39 (39%)
GG	37 (37%)
Maternal *FUT2* Genotype^c^	
AA	22 (22%)
AG	41 (41%)
GG	37 (37%)
Study Site	
Vancouver	40 (40%)
Edmonton	22 (22%)
Manitoba^d^	29 (29%)
Toronto	9 (9%)
Maternal Healthy Eating Index	72.1 ± 8.1
Maternal Artificially Sweetened Beverage Consumption During Pregnancy	50 (50%)

*SD* Standard deviation

^a^Data missing for two mother-infant dyads

^b^Data missing for three mother-infant dyads

^c^*FUT2* genotype indicates whether an individual is a non-secretor (AA) or secretor (AG or GG) of ABO histo-blood group antigens in other bodily fluids, such as on the gut mucosa or in breastmilk

^d^Includes Winnipeg and two rural sites, Morden and Winkler

Given the original selection criteria of this sub-cohort, we evaluated whether differences in early life exposures were observed relative to the rest of the CHILD Study cohort (*n* = 3,164). As expected, our sub-cohort included a higher proportion of infants whose mothers routinely consumed artificially sweetened beverages during pregnancy (50% vs. 29%, *p* < 0.001). This was accompanied by higher maternal BMI (26.7 ± 6.1 vs. 24.7 ± 5.4, *p* < 0.001), paternal BMI (28.5 ± 4.5 vs. 27.3 ± 4.7, *p* < 0.001), and sex-specific infant BMI z-scores (BMIz) at 3 months (females: 0.0 ± 0.9 vs. -0.3 ± 1.1, *p* = 0.049) and 1 year (females: 0.6 ± 1.3 vs. 0.1 ± 1.1, *p* = 0.004; males: 0.7 ± 1.1 vs. 0.2 ± 1.1, *p* = 0.005). Delivery by Cesarean (C)-section (36% vs. 25%; *p* = 0.018), lower rates of exclusive breastfeeding at 3 months (34% vs. 61%, *p* < 0.001), and earlier cessation of breastfeeding (7.8 ± 7.3 vs. 10.5 ± 6.8 months, *p* = 0.031) were also more common in this sub-cohort. This combination of BMI and early-life factors are dynamically related, with associations existing between high maternal BMI, increased likelihood of C-section delivery, and reduced breastfeeding duration, which may subsequently influence gut microbiome maturation [64–68]. Together, this indicates our sub-cohort represents a population with a greater propensity towards microbiome-disrupting exposures (i.e., C-section, formula feeding) and elevated BMI relative to the rest of the CHILD cohort.

### The bacterial and fungal gut microbiome exhibit divergent alpha diversity maturational patterns in the first year of life

**Fig. 1:**
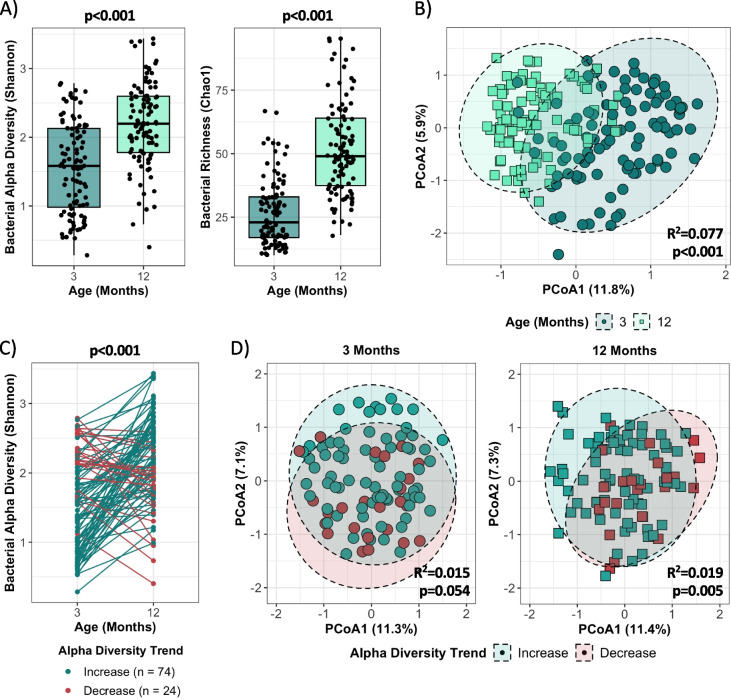
Divergent bacterial alpha diversity maturation patterns are observed in the first year of life. **A** Bacterial Shannon and Chao1 alpha diversity indices at 3 and 12 months of age, assessed by Mann–Whitney U test (3 months: *n* = 99, 12 months: *n* = 99). **B** Comparison of bacterial beta diversity using the Bray–Curtis dissimilarity index at 3 and 12 months, assessed by PERMANOVA (3 months: *n* = 99, 12 months: *n* = 99). Ellipses represent 95% CI. **C** Changes in bacterial alpha diversity (Shannon index) per individual from 3 to 12 months, assessed by paired t-test (increase: *n* = 74, decrease: *n* = 24; see Figure S2A for bacterial richness). **D** Comparison of bacterial beta diversity using the Bray–Curtis dissimilarity index by alpha diversity trend at 3 and 12 months, assessed by PERMANOVA (increase: *n* = 74, decrease: *n* = 24; see Figure S2B for bacterial richness trend). Ellipses represent 95% CI

Bacterial gut microbiome maturation patterns have been well-described, with increases in alpha diversity and decreases in beta diversity known to occur over the first years of life [16–20]. On average, we observed comparable overall changes in diversity metrics from 3 to 12 months of age in this cohort. Bacterial alpha diversity (Shannon) and richness (Chao1) increased from 3 to 12 months (Shannon: mean 1.55 ± 0.66 vs. 2.20 ± 0.61, *p* < 0.001; Chao1: mean 26.95 ± 13.12 vs. 52.13 ± 18.20, *p* < 0.001; Fig. 1A) and beta diversity decreased over the same period, indicating reductions in compositional dissimilarity of the microbiome between individuals at 12 months of age (*R*^2^ = 0.077, *p* < 0.001; Fig. 1B). While group-wise analyses have facilitated the identification of typical patterns of microbiome maturation in early life [16–20], our understanding of maturational patterns deviating from these descriptions is limited. We sought to investigate this by examining changes in bacterial alpha diversity and richness at the individual level and observed divergent patterns of microbiome maturation in the first year of life (Fig. 1C and Figure S2A, respectively). Although most infants (*n* = 74, or 75%) displayed the expected increase in bacterial alpha diversity from 3 to 12 months, 25% (*n* = 24) exhibited a decrease in alpha diversity over this period (*p* < 0.001; Fig. 1C). This divergence in microbiome maturation was also reflected in compositional differences (beta diversity) at 3 and 12 months, with alpha diversity trend explaining 1.5% and 1.9% of the variance, respectively (3 months: *p* = 0.054; 12 months: *p* = 0.005; Fig. 1D). Bacterial richness (Chao1) exhibited similar maturational patterns and compositional differences over the first year of life (Figure S2A-B).

**Fig. 2:**
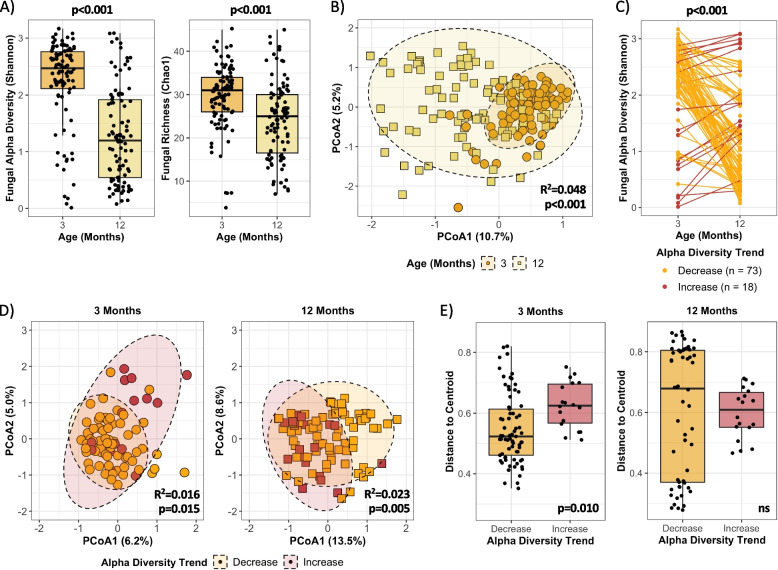
Divergent fungal alpha diversity maturation patterns are observed in the first year of life. **A** Fungal Shannon and Chao1 alpha diversity indices at 3 and 12 months of age, assessed by Mann–Whitney U test (3 months: *n* = 95, 12 months: *n* = 95). **B** Comparison of fungal beta diversity using the Bray–Curtis dissimilarity index at 3 and 12 months, assessed by PERMANOVA (3 months: *n* = 95, 12 months: *n* = 95). Ellipses represent 95% CI. **C** Changes in fungal alpha diversity (Shannon index) per individual from 3 to 12 months, assessed by paired t-test (decrease: *n* = 73, increase: *n* = 18; see Figure S3A for fungal richness). **D** Comparison of fungal beta diversity using the Bray–Curtis dissimilarity index by alpha diversity trend at 3 and 12 months, assessed by PERMANOVA (decrease: *n* = 73, increase: *n* = 18; see Figure S3B for fungal richness). Ellipses represent 95% CI. **E** Fungal beta dispersion by alpha diversity trend at 3 and 12 months, assessed by permutation test (decrease: *n* = 73, increase: *n* = 18)

In contrast to bacteria, descriptions of fungal gut microbiome maturation patterns are lacking, with inconsistent reports across studies regarding changes in fungal alpha and beta diversity in early life [3, 9, 21–28]. In this cohort, group-wise evaluations revealed that, on average, fungal alpha diversity (Shannon) and richness (Chao1) decreased from 3 to 12 months of age (Shannon: mean 2.23 ± 0.77 vs. 1.29 ± 0.83, *p* < 0.001; Chao1: mean 29.43 ± 7.65 vs. 24.18 ± 8.92, *p* < 0.001; Fig. 2A). Significant differences in beta diversity were also observed by age, with the composition of the mycobiome between individuals displaying opposite maturational patterns relative to bacteria, increasing in dissimilarity from 3 to 12 months (*R*^2^ = 0.048, *p* < 0.001; Fig. 2B). However, like bacteria, divergent alpha diversity and richness maturational trends were observed when assessed at the individual level (Fig. 2C and Figure S3A), with 80% (*n* = 73) of infants displaying a decrease and 20% (*n* = 18) increasing in fungal alpha diversity from 3 to 12 months (*p* < 0.001; Fig. 2C). Compositional differences at 3 and 12 months were also observed by fungal alpha diversity trend, explaining 1.6% and 2.3% of the variance, respectively (3 months: *p* = 0.015; 12 months: *p* = 0.005; Fig. 2D). The compositional differences observed by alpha diversity trend were further exemplified by significant differences in the distance to centroid, also known as beta dispersion, at 3 months (*p* = 0.010), but not at 12 months (Fig. 2E). No differences in beta dispersion were observed for bacterial alpha diversity trend. Divergent maturational patterns were also observed for fungal richness (Figure S3A-B), and previous work by our group has associated these changes with early-life BMI z-scores in this cohort [35].

Considering a substantial proportion of infants (20–25%) deviated from the predominant or “typical” pattern of change in bacterial and fungal alpha diversity observed in this sub-cohort, we sought to determine if “atypical” patterns of bacterial and fungal alpha diversity occurred in the same individual. However, we found that the infants that had an “atypical” (decreasing) bacterial alpha diversity trend generally had a “typical” (decreasing) fungal alpha diversity trend and vice versa, with only 2 infants displaying “atypical” trends for both bacteria and fungi. Overall, only 50 infants (56%) with data at both timepoints displayed “typical” trends for both bacterial and fungal alpha diversity. This highlights the importance of individual-level trajectory analyses in generating more nuanced understandings of bacterial and fungal microbiome maturation patterns in early life, particularly given the divergent alpha diversity trends observed were masked when performing group-wise analyses by age. For the remainder of this paper, alpha diversity trends will also be referred to as typical or atypical based on the predominant direction of change infants displayed in this sub-cohort: an increasing trend is typical for bacteria, whereas a decreasing trend is typical for fungi. While these definitions of “typical” and “atypical” are well-supported for bacterial alpha diversity in early life [16–20], our use of this terminology for fungi is specific to this sub-cohort and we cannot definitively say what is considered “typical” for fungal alpha diversity maturational patterns based on the current literature [3, 9, 21–28].

### Differences in taxonomic community structure are exhibited in infants with atypical bacterial or fungal alpha diversity trends

**Fig. 3:**
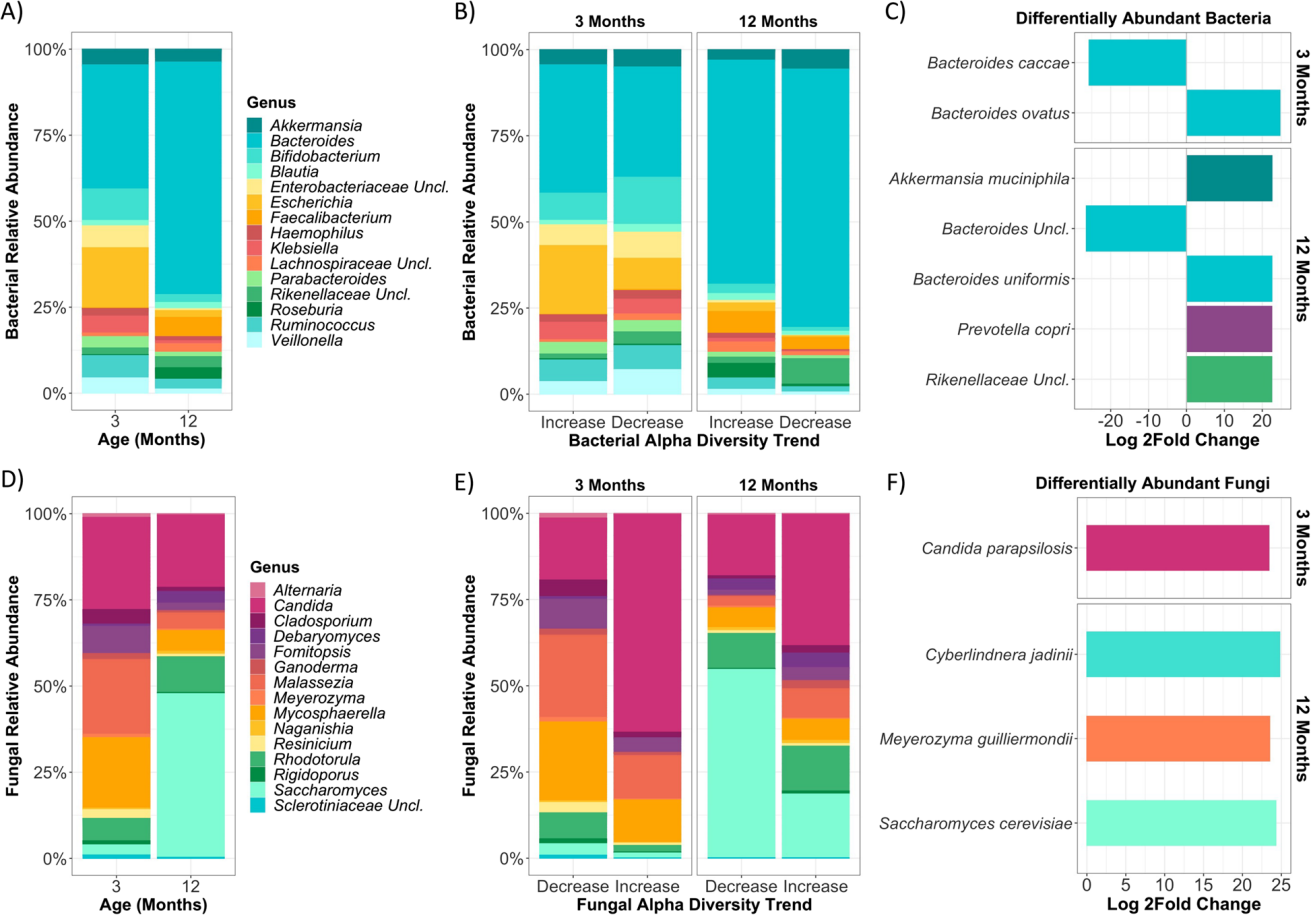
Differences in taxonomic structure are observed in infants with atypical bacterial and fungal alpha diversity trends in the first year of life. **A** Relative abundance of the 15 most abundant bacterial genera at 3 and 12 months (3 months: *n* = 99, 12 months: *n* = 99). **B** Relative abundance of the 15 most abundant bacterial genera by bacterial alpha diversity trend at 3 and 12 months (increase: *n* = 74, decrease: *n* = 24; see Figure S2C for bacterial richness trend). **C** Differentially abundant bacterial ASVs in the decreasing (atypical) bacterial alpha diversity trend relative to the increasing (typical) trend at 3 and 12 months (increase: *n* = 74, decrease: *n* = 24). **D** Relative abundance of the 15 most abundant fungal genera at 3 and 12 months (3 months: *n* = 95, 12 months: *n* = 95). **E** Relative abundance of the 15 most abundant fungal genera by fungal alpha diversity trend at 3 and 12 months (decrease: *n* = 73, increase: *n* = 18; see Figure S3C for fungal richness trend). **F** Differentially abundant fungal ASVs in the increasing (atypical) fungal alpha diversity trend relative to the decreasing (typical) trend at 3 and 12 months (decrease: *n* = 73, increase: *n* = 18)

Given over 40% of infants displayed atypical alpha diversity trends (for bacteria and/or fungi) and that community composition (beta diversity) differed by these trends, we next explored whether specific taxa were differentially associated with bacterial and fungal alpha diversity trends in the first year of life. First, we compared the relative abundances of the 15 most abundant bacterial and fungal genera by age, which represented 88.3 ± 14.6% and 85.3 ± 16.5% of the total community for bacteria at 3 and 12 months, respectively. The bacterial microbiome exhibited a more heterogenous taxonomic structure at 3 months, with *Bacteroides*, *Escherichia*, and *Bifidobacterium* being the most abundant genera, then shifted towards *Bacteroides* dominance at 12 months (Fig. 3A). In line with previous reports of broad compositional shifts occurring over the first year of life [16–20], we found significant differences in the relative abundances of all of the top 15 genera, except *Akkermansia*, *Haemophilus*, *Parabacteroides*, *Ruminococcus*, and unclassified *Rikenellaceae*, between 3 and 12 months (Table S2).

Next, we assessed the taxonomic structure by age and bacterial alpha diversity trend and observed smaller structural differences at both 3 and 12 months between infants with increasing and decreasing trends (Fig. 3B, Figure S4 and Table S2). At 3 months, the atypical or decreasing bacterial trend was associated with a significantly lower relative abundance of *Escherichia* (*p* = 0.013) relative to the typical or increasing trend; whereas, at 12 months, the atypical trend was associated with enrichment of *Bacteroides* (*p* = 0.016; Fig. 3B, Figure S4 and Table S2). To further probe what taxa were able to distinguish the typical alpha diversity trend from the atypical trend, we performed differential abundance analysis at the ASV level using DESeq2 [54]. At 3 months of age, infants that displayed an atypical bacterial alpha diversity trend had lower abundance of *Bacteroides caccae* (*p* < 0.001) and higher abundance of *Bacteroides ovatus* (*p* < 0.001; Fig. 3C). At 12 months, the atypical trend was associated with an elevated abundance of *Akkermansia muciniphila* (*p* < 0.001), *Bacteroides uniformis* (*p* < 0.001), *Prevotella copri* (*p* < 0.001), and unclassified *Rikenellaceae* (*p* < 0.001), alongside a lower abundance of unclassified *Bacteroides* (*p* < 0.001; Fig. 3C) relative to infants with a typical or increasing alpha diversity trend. Evidence of both the genus- and ASV-based taxonomic differences identified were also apparent when the relative abundance of the top 15 bacterial genera was assessed at the individual-level (Figure S4).

For fungi, the 15 most abundant genera represented 86.9 ± 10.5% and 93.3 ± 11.3% of the total community at 3 and 12 months, respectively. The mycobiome exhibited shared dominance of *Candida*, *Malassezia*, and *Mycosphaerella* at 3 months, then shifted towards *Saccharomyces* dominated communities at 12 months, with significant changes in the relative abundance of most of the top 15 genera exhibited over this time, except *Alternaria*, *Candida*, *Ganoderma*, *Resinicium*, and *Rigidoporus* (Fig. 3D and Table S3). Fungi exhibited more pronounced shifts in taxonomic structure when assessed by alpha diversity trend compared to bacteria, with the atypical or increasing trend being significantly enriched with *Candida* at 3 months relative to the typical trend (*p* < 0.001), alongside having lower abundances of *Malassezia* (*p* = 0.039), *Cladosporium* (*p* = 0.038), unclassified *Sclerotiniaceae* (*p* = 0.014), *Naganishia* (*p* = 0.006) and *Meyerozyma* (*p* < 0.001; Fig. 3E, Figure S5 and Table S3). At 12 months, the degree of *Candida* dominance was reduced, albeit remained more abundant than in infants with a typical fungal alpha diversity trend (*p* = 0.001; Fig. 3E, Figure S5 and Table S3). *Rigidoporus* was also significantly enriched in infants with an increasing fungal alpha diversity trend at 12 months (*p* = 0.033), while *Saccharomyces* (*p* = 0.005) and *Alternaria* (*p* = 0.001) were depleted (Fig. 3E, Figure S5 and Table S3). At the ASV level, infants with an increasing or atypical fungal alpha diversity trend had an enrichment of *Candida parapsilosis* (*p* < 0.001) at 3 months and *Cyberlindnera jadinii* (*p* < 0.001), *Meyerozyma guilliermondii* (*p* < 0.001), and specific *Saccharomyces cerevisiae* ASVs (*p* < 0.001) at 12 months relative to those with a decreasing trend (Fig. 3F). Again, these findings were supported by individual-level assessment of the relative abundance of the top 15 genera by alpha diversity trend and age (Figure S5). Altogether, this analysis revealed that dynamic shifts in bacterial and fungal community structure underlie patterns of alpha diversity change in early life.

### Atypical alpha diversity trends are associated with altered inter-kingdom co-occurrence dynamics

Next, we employed co-occurrence network analyses at the species level to examine inter-kingdom dynamics between bacteria and fungi. These methods facilitate the prediction of potential hub species and rank their positional importance within the ecosystem [69]. Metrics applied in ecological network analysis are also used to characterize and compare the organization and functioning of ecosystems, facilitating the inference of multi-trophic interactions amongst species [70]. Infants were evaluated based on the combination of alpha diversity trends they exhibited for both bacteria and fungi. Overall, 89 infants had adequate data for both timepoints and kingdoms. Of these, 50 (56%) infants displayed a typical or inverse overall trend between bacterial and fungal alpha diversity with bacterial alpha diversity increasing and fungal alpha diversity decreasing from 3 to 12 months, 21 (24%) had an atypical decrease in bacterial alpha diversity, 16 (18%) had an atypical increase in fungal alpha diversity, and 2 (2%) displayed an atypical trend for both bacteria and fungi. Infants with an atypical trend for both bacterial and fungal alpha diversity (*n* = 2) were omitted from subsequent analyses due to sample size limitations.

**Fig. 4:**
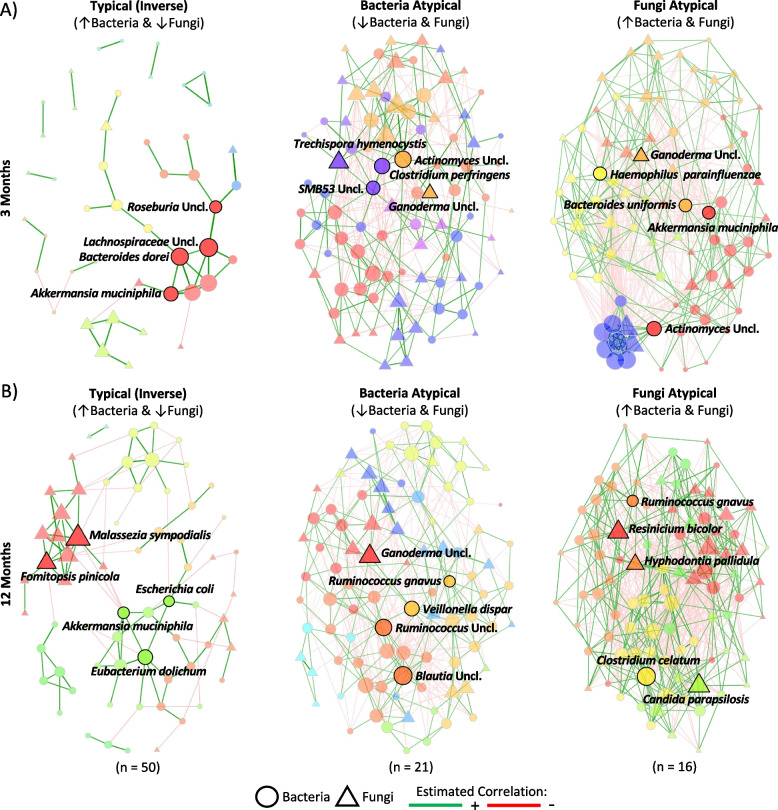
Structural differences in inter-kingdom co-occurrence networks exist between infants with typical (inverse) and bacteria or fungi atypical overall alpha diversity trends in the first year of life. Inter-kingdom correlation networks of bacterial and fungal species based on overall alpha diversity trends at **A** 3 and **B** 12 months. Infants were classified into overall alpha diversity relationships based on the combination of alpha diversity trends they exhibited for bacteria and fungi. A typical inverse trend was characterized by increasing bacterial and decreasing fungal alpha diversity (*n* = 50); a bacteria atypical trend was characterized by decreasing bacterial and fungal alpha diversity (*n* = 21); and a fungi atypical trend was characterized by increasing bacterial and fungal alpha diversity (*n* = 16). Networks were generated using the fast greedy clustering algorithm with a minimum Pearson correlation coefficient threshold of ± 0.4. Positive correlations are displayed in green and negative correlations in red. Bacterial species are represented by circles and fungal species by triangles. Node size was determined based on degree centrality. Hub taxa are those with the highest betweenness centrality and are labelled with their shape perimeter bolded. Shape colour represents clusters of species more likely to co-occur with one another than with species from outside of these modules. Pair-wise comparisons of network measures were calculated using 5,000 permutations (see Table S4)

Inter-kingdom network analyses revealed distinct differences in the structure of co-occurrence networks between typical (inverse) and atypical (bacteria or fungi) overall alpha diversity trends at both 3 and 12 months (Fig. 4 and Table S4). This was reflected in significant differences between various network measures of centrality and the emergence of distinct hub taxa in each network (Fig. 4 and Table S4), highlighting the unique inter-kingdom co-occurrence dynamics exhibited across all three patterns of microbiome maturation at both timepoints. In the co-occurrence network for the typical (inverse) bacterial and fungal alpha diversity trends at 3 months, members of the core infant gut microbiome, *Roseburia* spp., unclassified *Lachnospiraceae*, *Bacteroides dorei*, and *Akkermansia muciniphila* were identified as hub taxa (Fig. 4A), forming a functional cluster consistent with the metabolic cross-feeding dynamics involved in butyrate metabolism in these ecosystems [71–74]. In contrast, atypical networks displayed a higher density of co-occurrence relationships, with a mix of core and opportunistic microbes emerging as hub taxa (Fig. 4). These hub taxa were dispersed across more functional clusters compared to the networks obtained from the typical alpha diversity trends, but some core members were maintained as hubs in the fungi atypical network at 3 months (e.g., *A. muciniphila*), which likely reflects the expected increase in bacterial alpha diversity observed in these communities (Fig. 4). This is further supported by the higher natural connectivity (robustness) of the fungi atypical network compared to the bacterial atypical network (*p* = 0.017), being more comparable to the typical network (Table S4), suggesting that an atypical decrease in bacterial alpha diversity may have different and stronger effects on community dynamics than an increase in fungal alpha diversity.

When comparing network measures of centrality at 3 months, the atypical bacterial alpha diversity trend network exhibited differences in betweenness centrality (network members that ‘bridge’ between nodes; *p* = 0.013) and Eigenvector centrality (level of influence of a node within a network; *p* = 0.003) relative to the typical inverse network (Table S4). Similarly, networks from infants exhibiting an atypical fungal trend differed in terms of degree (total number of edges or links between nodes; *p* = 0.003), closeness centrality (shortest path between nodes; 0.013), betweenness centrality (*p* = 0.041), and Eigenvector centrality (*p* = 0.013) compared to the typical trend, with comparable differences also emerging between bacteria and fungi atypical networks (Table S4). Centrality metrics denote how important a node is for the connectivity and interactions within the network. That is, a node of high centrality is required for paths leading to other nodes, and consequently, have a greater likelihood of being involved in the network’s predicted food chains [75]. Through this lens, this analysis suggests that typical microbiome maturation during infancy favours hubs of higher positional importance within the networks.

At 12 months, the network from infants with typical alpha diversity trends maintained functional clusters of core bacterial microbiome members (i.e., *A. muciniphila* and *Eubacterium dolichum*) identified as hub taxa. In contrast, both atypical trends continued to display more densely connected networks and contained a combination of hub taxa that were present at 3 months (i.e., *Clostridium, Ganoderma*) or are core microbiome members (i.e., *Blautia*, *Ruminococcus*; Fig. 4). *Candida parapsilosis* also emerged as a hub in the fungi atypical network at 12 months (Fig. 4B), consistent with the significantly higher relative abundance of *Candida* in infants with an atypical fungal alpha diversity trend at 3 and 12 months relative to those with a typical trend (Fig. 3E, F and Table S3). Network metrics at 12 months showed very similar results to those at 3 months (Table S4). The typical network exhibited differences in degree (*p* = 0.002) and betweenness centrality (*p* = 0.027) relative to the network from infants with an atypical bacterial alpha diversity trend, as well as differences in degree (*p* = 0.027) and Eigenvector centrality (*p* = 0.008) when compared to the network for those with an atypical fungal alpha diversity trend (Table S4). Meanwhile, the two atypical networks exhibited differences across degree (*p* < 0.001), betweenness (*p* = 0.003), closeness (*p* = 0.003), and Eigenvector centrality (*p* = 0.013; Table S4). This further supports the idea that the typical alpha diversity trend network favours highly centralized nodes within trophic webs. In parallel, these consistent differences in network structure and hub taxa between typical and atypical alpha diversity trends at 3 and 12 months may indicate either earlier or delayed community transitions in infants with atypical bacterial or fungal alpha diversity trends.

To determine whether the observed co-occurrence dynamics were exclusively a function of inter-kingdom influences, we generated networks for each kingdom in isolation (Figures S6-7). In both cases, the structure of the typical vs. atypical bacterial or fungal networks mirrored those of the inter-kingdom networks, with fewer taxa passing the correlation threshold in the typical networks (Figures S6-7 and Tables S5-6). To evaluate if the differences in the network structures were a function of unequal sample sizes between the three groups, we compared the inter-kingdom networks between a typical inverse relationship for bacterial and fungal alpha diversity (*n* = 50) and all the atypical patterns combined (decreasing bacterial alpha diversity, increasing fungal alpha diversity, or both; *n* = 39). While this provided a level of control for the number of species passing the correlation threshold (i.e., the number of species that pass the Pearson threshold is proportional to the number of samples evaluated), making it more comparable between the typical and atypical networks, many of the observed differences in network properties and hub taxa persisted (Figure S8 and Table S7). Together, this suggests atypical shifts in alpha diversity in the first year of life are associated with altered microbial co-occurrence dynamics when compared to infants with a typical inverse overall trend between bacterial and fungal alpha diversity and that these changes may reflect differences in the rate of gut microbiome maturation.

### Bacterial and fungal alpha diversity trends are associated with multi-kingdom dynamics, FUT2 secretor status, and other known microbiome-modifying factors in early life

**Fig. 5:**
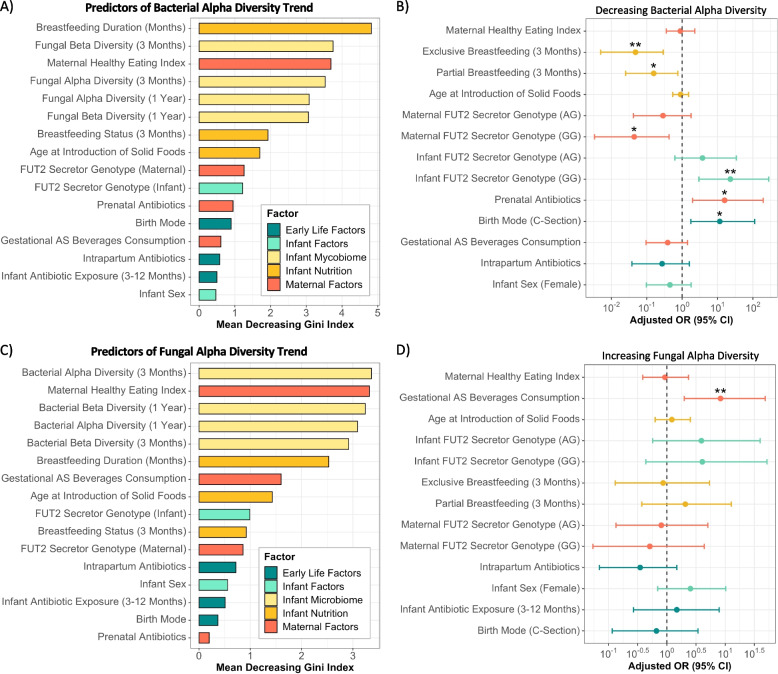
Multi-kingdom dynamics, maternal and infant nutrition, delivery mode, and antibiotic exposure are associated with atypical bacterial and fungal alpha diversity trends. **A** Predictors of bacterial alpha diversity trend identified by random forest using 10-fold cross-validation, 500 trees, and 1,000 permutations (increase: *n* = 63, decrease: *n* = 22). The increasing alpha diversity trend was set as the reference level. **B** Multivariable logistic regression identifying associations between early life, infant, and maternal factors and a decreasing (atypical) bacterial alpha diversity trend (increase: *n* = 70, decrease: *n* = 23). The increasing alpha diversity trend was set as the reference level. **C** Predictors of fungal alpha diversity trend identified by random forest using 10-fold cross-validation, 500 trees, and 1,000 permutations (decrease: *n* = 68, increase: *n* = 17). The decreasing alpha diversity trend was set as the reference level. **D** Multivariable logistic regression identifying associations between early life, infant, and maternal factors and an increasing (atypical) fungal alpha diversity trend (decrease: *n* = 71, increase: *n* = 17). The decreasing alpha diversity trend was set as the reference level. AS, artificially sweetened; GI, Gini index; AG and GG *FUT2* secretor genotypes are secretors, reference level AA genotype are non-secretors; ~ *p* < 0.1; *p < 0.05; **p < 0.01

We next sought to understand whether early-life, infant, maternal, nutritional, and ecological factors were linked to the divergent microbiome maturation patterns we observed in the first year of life. First, we employed random forests to determine the factors that were predictive of whether an infant displayed an increasing or decreasing alpha diversity trend. For the bacterial alpha diversity trend, breastfeeding duration (GI = 5.06), fungal alpha and beta diversity at 3 (alpha: GI = 3.55; beta: GI = 3.92) and 12 months (alpha: GI = 2.95; beta: GI = 2.77), and maternal healthy eating index (GI = 3.73) had the greatest discriminatory power, followed by breastfeeding status at 3 months (GI = 1.78), age at introduction of solid foods (GI = 1.76), and maternal (GI = 1.46) and infant (GI = 1.04) *FUT2* secretor genotypes (Fig. 5A). The directionality of these relationships was then explored using logistic regression, while considering the confounding effects of infant, early-life, and maternal factors. Infants who were breastfed at 3 months, either partially (OR = 0.16, CI: 0.03–0.75, *p* = 0.029) or exclusively (OR = 0.05, CI: 0.00–0.29, *p* = 0.003), were less likely to have an atypical or decreasing bacterial alpha diversity trend (Fig. 5B and Table S8). Maternal *FUT2* secretor genotype displayed a similar inverse relationship for the homozygous (GG) allele (OR = 0.04, CI: 0.00–0.43, *p* = 0.010), whereas the homozygous (GG) allele in infants was positively associated with an atypical bacterial alpha diversity trend (OR = 23.02, CI: 2.96–280.06, *p* = 0.006; Fig. 5B and Table S8). Delivery via C-section also emerged as positively associated with a decreasing bacterial alpha diversity trend (OR = 11.57, CI: 1.76–112.13, *p* = 0.019), alongside prenatal antibiotics exposure (OR = 15.80, CI: 1.96–194.97, *p* = 0.017; Fig. 5B and Table S8).

The fungal alpha diversity trend was similarly predicted by multi-kingdom dynamics, including bacterial alpha and beta diversity at 3 (alpha: GI = 3.66; beta: GI = 3.10) and 12 months (alpha: GI = 3.18; beta: GI = 2.99) of age, maternal healthy eating index (GI = 3.23), and breastfeeding duration (GI = 2.45; Fig. 5C). Maternal consumption of artificially sweetened beverages during gestation (GI = 1.47), age at introduction of solid foods (GI = 1.32), infant *FUT2* secretor genotype (GI = 1.06), and breastfeeding status at 3 months (GI = 0.97) also emerged as strong predictors (Fig. 5C). Logistic regression revealed maternal consumption of artificially sweetened beverages during gestation was positively associated with an atypical or increasing fungal alpha diversity trend (OR = 8.32, CI: 1.98–48.59, *p* = 0.008; Fig. 5D and Table S9), but no other significant associations were observed. Together, our analyses suggest that the role of known microbiome-modifying factors, such as breastfeeding duration and birth mode, in the developmental patterns of the bacterial microbiome may not be as influential on fungal microbiome maturation. This work further revealed that multi-kingdom diversity metrics are associated with bacterial and fungal alpha diversity trends, and maternal and infant *FUT2* secretor genotypes are associated with bacterial alpha diversity, prompting for further explorations of the effects of ecological interactions between bacteria and fungi, as well as secretor status, on microbiome establishment.

### Atypical bacterial and fungal alpha diversity trends are associated with metabolomic shifts in urine at three months of age

**Fig. 6:**
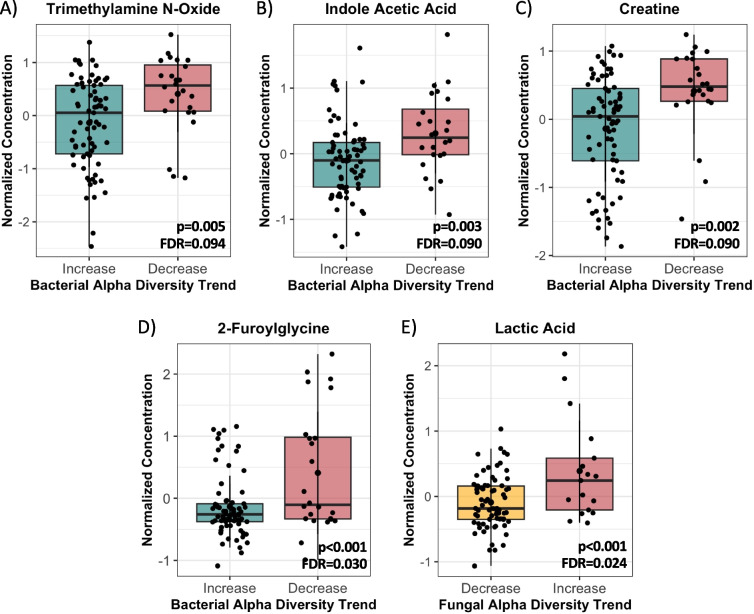
Atypical bacterial and fungal alpha diversity trends are associated with functional differences reflected in urine metabolites at 3 months. Normalized concentrations of **A** trimethylamine N-oxide (TMAO), **B** indole acetic acid (IAA), **C** creatine, and **D** 2-furoylglycine by bacterial alpha diversity trend (increase: *n* = 74, decrease: *n* = 24) and **E** lactic acid by fungal alpha diversity trend (decrease: *n* = 73, increase: *n* = 18) at 3 months observed in urine, assessed by t-test. Metabolite concentrations were normalized using the median, log-transformed, and pareto-scaled (mean-centered and divided by the square root of the standard deviation of each metabolite) and a false discovery rate (FDR) cutoff of 0.1 was applied. No significant differences in urine metabolite concentrations were observed at 12 months

To investigate whether the differences observed in taxonomic community structure between alpha diversity patterns were associated with functional changes, we performed untargeted urine metabolomics at 3 and 12 months of age. Metabolite evaluation in urine has the advantage of revealing markers of physiological or pathological host–microbe interactions, as microbiome-derived products can be excreted renally [76]. We identified differences in the concentration of specific urine metabolites between the typical and atypical alpha diversity trends for both bacteria and fungi at 3, but not 12, months of age (Fig. 6). Infants with an atypical or decreasing bacterial alpha diversity trend exhibited significant enrichment of trimethylamine N-oxide (TMAO; *p* = 0.005; Fig. 6A), indole acetic acid (IAA; *p* = 0.003; Fig. 6B), creatine (*p* = 0.002; Fig. 6C), and 2-furoylglycine (*p* < 0.001; Fig. 6D) relative to those with a typical or increasing bacterial alpha diversity trend. For fungi, an atypical or increasing alpha diversity trend was associated with higher concentrations of lactic acid (*p* < 0.001; Fig. 6E). Given most metabolites (*n* = 102) remained unchanged when assessed by either the bacterial or fungal alpha diversity trend, this suggests that compositional changes in the gut microbiome likely did not translate to systemic host functional shifts, but investigations of the serum and stool metabolomes could help confirm this. However, the increases observed in the concentration of metabolites with known microbial origins (e.g., TMAO, IAA, lactic acid) [77–79] implies these changes in gut microbial composition may still have functional consequences on the host.

## Discussion

A signature feature of primary ecological succession in bacterial communities is an increase in alpha diversity, propelled mainly by non-stochastic, niche-driven effects [80]. These predictable patterns of primary succession have also been observed in the bacterial gut microbiome of infants over the first year of life, based on group-wise comparisons of diversity metrics across early-life timepoints [16–20]. By evaluating ecological shifts at the individual level, we identified divergent trajectories of gut microbiome maturation across 100 Canadian infants based on changes in bacterial and fungal alpha diversity per individual from 3 to 12 months of age, which were masked when performing group-based analyses. These trajectories occurred in over 40% of infants and were characterized by distinct differences in community composition, inter-kingdom co-occurrence dynamics, and the abundance of select microbially-derived urine metabolites, suggestive of variable rates of microbiome maturation. Factors known to be involved in directing early life bacterial microbiome maturation, such as breastfeeding and delivery mode [19, 20, 57], were associated with these patterns for bacteria, but not fungi. Together, this work highlights the important knowledge gaps created when microbiome research focuses exclusively on group-based, single-kingdom, and/or cross-sectional analyses.

Successional patterns of infant gut microbiome maturation have been well-described for the bacterial microbiome [16–20], while only a handful of reports on fungal microbiome maturation patterns exist and most are limited by small sample sizes [3, 9, 21–28]. In this sub-cohort, the overall changes in the taxonomic structure of the bacterial and fungal gut microbiome from 3 to 12 months largely followed what has been previously reported [3, 9, 16–28], but differences emerged in infants with atypical bacterial or fungal alpha diversity trends, suggesting alterations in the arrival times of specific microbes, availability of appropriate niches, or initiation of ecosystem transitions. For example, *Ruminococcus gnavus* has been identified as marker of microbiome immaturity [20, 81], but emerged as a hub in both atypical networks at 12 months. Similarly, *Candida* is typically dominant very early in fungal microbiome maturation [3, 21, 22, 27], but infants with an atypical fungal alpha diversity trend maintained a high abundance of *Candida* and failed to transition to *Saccharomyces-*predominant communities over the first year of life, with *Candida parapsilosis* being identified as a hub taxon in the atypical co-occurrence network at 12 months. Transition towards communities enriched with *Saccharomyces* has been previously linked to the introduction of solid foods [3, 9]; however, this factor was not significantly associated with an atypical fungal alpha diversity trend in our study, suggesting more complex ecosystem dynamics may underly this incomplete compositional transition.

Our investigations of inter-kingdom dynamics revealed stark differences in the structure of co-occurrence networks between infants with typical and atypical maturational patterns at both 3 and 12 months of age, regardless of whether the atypical trends were driven by changes in bacteria or fungi. Networks for the typical (inverse) bacterial and fungal alpha diversity trend displayed defined functional clusters with few taxa passing the correlation threshold, which increased in complexity from 3 to 12 months. In contrast, both atypical trends exhibited densely connected networks that were structurally comparable between timepoints. These differences may indicate reduced microbiome maturity and lack of successional progression in infants with an atypical bacterial or fungal alpha diversity trend, given densely connected ecosystems are more vulnerable to disturbances [82]. In contrast, more competitive microbial community dynamics with fewer taxa passing the correlation threshold, such as the ones observed for the typical alpha diversity trends, are associated with increased community stability and maturity [82]. This is further emphasized by the increase in modularity in the typical networks from 3 to 12 months, evidenced by the distinct functional clusters separated by negative co-occurrence relationships, suggestive of the formation of sub-communities driven by ecological processes such as habitat filtering or niche occupation [83]. Meanwhile, the differences in centrality measures across each network highlights the distinct community dynamics and hubs, or most central taxa, underlying the varied patterns of microbiome maturation. Ultimately, the observed inter-kingdom co-occurrence dynamics suggest atypical shifts in bacterial or fungal alpha diversity in the first year of life may limit the ability of the microbiome to form resilient, stable communities, potentially due to overly cooperative dynamics that prevent or delay subsequent successional steps from occurring. This is supported by experimental evidence from eco-evolutionary models, showing that evolution limits cooperation among microbial community members, as this increases dependency on species that may not be present and renders less productive communities [84].

Functionally, select metabolites with known microbial origins were found to be elevated at 3 months in infants with an atypical bacterial or fungal alpha diversity trend, suggesting these divergent maturational patterns may have important functional implications. First, the higher creatine levels observed in those with an atypical bacterial trend support our hypothesis that these infants may be experiencing delayed microbiome maturation, as reductions in creatine have been associated with microbial colonization and microbiome maturity in both animal models and humans, explained by microbial involvement in creatine elimination [85–88]. Meanwhile, the enrichment of metabolic by-products of various microbial metabolic pathways, including TMAO (protein catabolism) and IAA (tryptophan catabolism) in the atypical bacterial trend and lactic acid (sugar anabolism) in the atypical fungal trend, suggest these alterations may have broad functional effects on the host. For example, enrichment of IAA in infants with an atypical bacterial alpha diversity trend may reflect greater tryptophan metabolism by the IAA-producer, *Bacteroides ovatus* [89], whose relative abundance is significantly higher in the atypical bacterial trend at 3 months. This could translate to broad physiological influences on the host, as IAA is involved in immune homeostasis, gut-brain communication, regulating epithelial integrity, and host gene expression [77, 89–91]. In contrast, the accumulation of lactic acid in the atypical fungal alpha diversity trend may indicate the absence of lactate-consuming, butyrate-producing strains in the microbiome of these infants, such as *Roseburia*, which could have downstream impacts on gut epithelium integrity due to the role of butyrate in colonocyte health [92–95]. Although these metabolic changes are not maintained longitudinally, being observed at 3 months only, it is possible that they may still be influential given the rapid developmental processes and pronounced influence of host-microbiome crosstalk during this early-life critical window [31, 32].

Current understandings of the factors influencing gut microbiome maturation patterns in early life are based on the bacterial microbiome. Our study found that the factors related to the bacterial alpha diversity trend are largely consistent with the literature, including the effects of breastfeeding, antibiotics, and mode of birth [19, 20, 57]. Yet, we also identified a role of both maternal and infant *FUT2* secretor genotype in microbiome maturational trajectories, with the directionality of the association changing depending on whether the mother or infant was a homozygous (GG) secretor. This intriguing finding may reflect differential influences of maternal vs. infant secretor status on microbial metabolism and gut physiology, as the secretion of ABO histo-blood group antigens in breast milk and on the gut mucosa act as carbohydrate substrates for microbes, and thus, may favour certain microbes occupying specific geospatial niches [8, 96]. For example, secretor mothers produce fucosylated human milk oligosaccharides (HMOs) in breastmilk that select for the expansion of HMO-utilizing bifidobacteria, and subsequently, encourage cooperative microbial cross-feeding dynamics in these communities [8, 96, 97]. This has been associated with increased alpha diversity in breastfed infants [8, 96, 97], consistent with our results. In contrast, infant secretors express these carbohydrate groups on the mucus lining of the gut, which may influence microbial community composition by favoring mucosa-associated microbes or mucin degraders, such as *Akkermansia muciniphila* [98]. This could explain the expansion of *Akkermansia muciniphila* observed at 12 months in infants with an atypical bacterial alpha diversity trend, as this trend was positively associated with infant *FUT2* secretor status. Given the variability in associations previously reported between secretor status and microbiome composition [97, 99, 100], this relationship is likely complex, but our finding highlights the need to consider the influence of both maternal and infant genetic factors on microbiome maturation patterns in early life.

Unlike the divergent bacterial microbiome maturational trajectories, fungal alpha diversity trend was largely not associated with known bacterial microbiome-modifying factors, apart from exposure to artificial sweeteners [36], suggesting fungal colonization may be directed by factors beyond commonly studied pre- and post-natal exposures. Instead, we found alpha and beta diversity metrics of the opposing kingdom to be robust predictors of alpha diversity trend directionality. This highlights the importance of multi-kingdom microbial interactions during infant microbiome assembly, as within-ecosystem dynamics beyond bacteria may have differential and stronger influences on microbial colonization patterns than external factors. For example, a recent ecological analysis by Rao et al*.* revealed that *Candida albicans* dictated early microbial assembly by inhibiting *Escherichia* and *Klebsiella* colonization, while its own expansion was prevented by *Staphylococcus* [10]. Considering the substantial relative abundance of *Candida* in infants with an atypical fungal alpha diversity trend at both 3 and 12 months, it is possible that similar inter-kingdom ecosystem dynamics may underly these different maturational patterns. Together, these findings call for the inclusion of additional microbiome members in studies on early-life gut microbiome maturation and highlight the limitations of generalizing our understandings of factors influencing bacterial colonization patterns to other kingdoms.

The main strength of our study is the incorporation of multi-kingdom data and individual-level longitudinal analyses to add improved resolution to our understanding of bacterial and fungal gut microbiome maturation patterns in early life. By evaluating bacterial and fungal members of the gut microbiome together, we were able to identify the important influence of inter-kingdom factors on microbiome maturation and generate clearer understandings of the differences in microbial co-occurrence dynamics between typical and atypical maturation patterns. However, while the network analyses used to generate these findings are informative and hypothesis-generating, it is important to note that they inherently come with limitations, particularly when based on compositional vs. absolute data, and the biological interactions inferred should be reproduced in other cohorts and corroborated experimentally to confirm their relevance. Further, although our study is constrained by sample size, this limitation simultaneously highlights the prevalence of diverging microbiome maturation patterns in early life, calling for greater research attention.

Future work should focus on the incorporation of repeated microbiome measures and additional functional analyses (e.g., fecal metabolomics, immune markers) to determine if the atypical alpha diversity trends observed vary within or extend beyond the first year of life and clarify the functional effects of atypical trajectories of microbiome maturation on the host. This could be further strengthened through the incorporation of metagenomic analyses to help overcome the limitations of amplicon-based sequencing, particularly by providing broader functional measures, improved bacterial taxonomic assignment, and enabling the interrogation of other microbiome members (e.g., bacteriophage, viruses, Archaea) and how they contribute to gut microbial ecosystem dynamics. In parallel, longitudinal data on health outcomes in childhood and adolescence would provide important insights into the developmental implications of these divergent maturational patterns, which are unclear in this work due to our early-life focus. Despite these limitations, our research clearly highlights the pitfalls of reductionist (e.g., bacteria only) and exclusively group-based analytical approaches in gut microbiome research, which have a greater propensity to mask more complex ecosystem dynamics and yield incomplete narratives.

Overall, our findings suggest atypical patterns of bacterial and fungal gut microbiome succession are more common than previously considered, and that these patterns may be indicative of delayed or variable rates of microbiome maturation. Analyses in large, longitudinal cohorts containing data on health outcomes and repeated microbiome measures will be imperative to determine whether the atypical microbiome maturation patterns observed have long-term consequences. Our work also determined that while the mycobiome plays an important role in bacterial microbiome establishment during early life, the factors influencing fungal microbiome maturation differ from those commonly reported for the bacterial microbiome and remain underexplored. It may be the case that the mycobiome is more strongly influenced by stochastic factors, within-ecosystem dynamics, or other social and environmental factors (e.g., cultural differences in diet, geography, seasonality). Future work should seek to better delineate the differential influences of early-life exposures on the bacterial vs. fungal microbiome, as well as how inter-kingdom dynamics contribute to gut colonization patterns. Ultimately, understanding the ecological and host-derived processes behind microbial primary succession may be useful within restoration and conservation frameworks aimed at improving the health trajectories of children at risk of or already displaying early-life microbiome alterations.

### Supplementary Information

**Additional file 1: Table S1.** 16S and ITS2 read counts before and after sequence processing with the *DADA2* pipeline (related to Figure S1). **Table S2.** Differences in CLR-transformed abundance of the top 15 bacterial genera by infant age and bacterial alpha diversity trend (related to Fig. 3A-B and Figure S4). **Table S3.** Differences in CLR-transformed abundance of the top 15 fungal genera by infant age and fungal alpha diversity trend (related to Fig. 3D-E and Figure S5). **Table S4.** Pair-wise comparison of typical (inverse), bacteria atypical, and fungi atypical inter-kingdom microbial co-occurrence network properties at 3 and 12 months (related to Fig. 4). **Table S5.** Bacterial co-occurrence network properties between typical and atypical alpha diversity trends at 3 and 12 months (related to Figure S6). **Table S6.** Fungal co-occurrence network properties between typical and atypical alpha diversity trends at 3 and 12 months (related to Figure S7). **Table S7.** Inter-kingdom co-occurrence network properties between typical and atypical (bacteria, fungi, or both) alpha diversity trends at 3 and 12 months (related to Figure S8). **Table S8.** Logistic regression statistics between maternal, infant, and early-life factors and bacterial alpha diversity trend (related to Fig. 4B). **Table S9.** Logistic regression statistics between maternal, infant, and early-life factors and fungal alpha diversity trend (related to Fig. 4D). **Figure S1.** 16S and ITS2 sequencing depth and sample composition (related to Table S1). **Figure S2.** Divergent bacterial richness maturation patterns are observed in the first year of life (related to Fig. 1). **Figure S3.** Divergent fungal richness maturation patterns are observed in the first year of life (related to Fig. 2). **Figure S4.** Individual-level taxonomic differences between infants with an increasing vs. decreasing bacterial alpha diversity trend at 3 and 12 months (related to Fig. 3 and Table S2). **Figure S5.** Individual-level taxonomic differences between infants with a decreasing vs. increasing fungal alpha diversity trend at 3 and 12 months (related to Fig. 3 and Table S3). **Figure S6.** Differences in bacterial co-occurrence networks are observed between increasing and decreasing alpha diversity trends at 3 and 12 months (related to Fig. 4 and Table S5). **Figure S7.** Differences in fungal co-occurrence networks are observed between increasing and decreasing alpha diversity trends at 3 and 12 months (related to Fig. 4 and Table S6). **Figure S8.** Differences in inter-kingdom co-occurrence networks are observed between infants with a typical (inverse) bacterial and fungal alpha diversity trend and atypical changes in bacterial, fungal, or both alpha diversity trends at 3 and 12 months (related to Fig. 4 and Table S7).

## Data Availability

Demultiplexed bacterial and fungal sequences are deposited on the NCBI sequence read archive (PRJNA624780 and PRJNA814728, respectively). All code (R scripts) has been deposited at: https://github.com/ArrietaLab/CHILD_InfantGutMicrobiomeEcology.
