# Supplementary material for: Divergent maturational patterns of the infant bacterial and fungal gut microbiome in the first year of life are associated with inter-kingdom community dynamics and infant nutrition
Source: Microbiome. 2024 Feb 7;12:22. doi: 10.1186/s40168-023-01735-3 (PMC10848358; doi:10.1186/s40168-023-01735-3)
Supplement: Supplementary file 2 — Additional file 1: Table S1. 16S and ITS2 read counts before and after sequence processing with the DADA2 pipeline (related to Figure S1). Table S2. Differences in CLR-transformed abundance of the top 15 bacterial genera by infant age and bacterial alpha diversity trend (related to Fig. 3A-B and Figure S4). Table S3. Differences in CLR-transformed abundance of the top 15 fungal genera by infant age and fungal alpha diversity trend (related to Fig. 3D-E and Figure S5). Table S4. Pair-wise comparison of typical (inverse), bacteria atypical, and fungi atypical inter-kingdom microbial co-occurrence network properties at 3 and 12 months (related to Fig. 4). Table S5. Bacterial co-occurrence network properties between typical and atypical alpha diversity trends at 3 and 12 months (related to Figure S6). Table S6. Fungal co-occurrence network properties between typical and atypical alpha diversity trends at 3 and 12 months (related to Figure S7). Table S7. Inter-kingdom co-occurrence network properties between typical and atypical (bacteria, fungi, or both) alpha diversity trends at 3 and 12 months (related to Figure S8). Table S8. Logistic regression statistics between maternal, infant, and early-life factors and bacterial alpha diversity trend (related to Fig. 4B). Table S9. Logistic regression statistics between maternal, infant, and early-life factors and fungal alpha diversity trend (related to Fig. 4D). Figure S1. 16S and ITS2 sequencing depth and sample composition (related to Table S1). Figure S2. Divergent bacterial richness maturation patterns are observed in the first year of life (related to Fig. 1). Figure S3. Divergent fungal richness maturation patterns are observed in the first year of life (related to Fig. 2). Figure S4. Individual-level taxonomic differences between infants with an increasing vs. decreasing bacterial alpha diversity trend at 3 and 12 months (related to Fig. 3 and Table S2). Figure S5. Individual-level taxonomic differences between infants [file 40168_2023_1735_MOESM1_ESM.docx]

**Divergent maturational patterns of the infant bacterial and fungal gut microbiome in the first year of life are associated with inter-kingdom community dynamics and infant nutrition**

**(Mercer *et al.*)**

**Supplementary Tables & Figures:**

**Table S1. 16S and ITS2 read counts before and after sequence processing with the *DADA2* pipeline (related to Figure S1).**

**Table S2. Differences in CLR-transformed abundance of the top 15 bacterial genera by infant age and bacterial alpha diversity trend (related to Figure 3A-B and Figure S4).**

**Table S3. Differences in CLR-transformed abundance of the top 15 fungal genera by infant age and fungal alpha diversity trend (related to Figure 3D-E and Figure S5).**

**Table S4. Pair-wise comparison of typical (inverse), bacteria atypical, and fungi atypical inter-kingdom microbial co-occurrence network properties at 3 and 12 months (related to Figure 4).**

**Table S5. Bacterial co-occurrence network properties between typical and atypical alpha diversity trends at 3 and 12 months (related to Figure S6).**

**Table S6. Fungal co-occurrence network properties between typical and atypical alpha diversity trends at 3 and 12 months (related to Figure S7).**

**Table S7. Inter-kingdom co-occurrence network properties between typical and atypical (bacteria, fungi, or both) alpha diversity trends at 3 and 12 months (related to Figure S8).**

**Table S8. Logistic regression statistics between maternal, infant, and early-life factors and bacterial alpha diversity pattern (related to Figure 4B).**

**Table S9. Logistic regression statistics between maternal, infant, and early-life factors and fungal alpha diversity pattern (related to Figure 4D).**

**Figure S1. 16S and ITS2 sequencing depth and sample composition (related to Table S1).**

**Figure S2. Divergent bacterial richness maturation patterns are observed in the first year of life (related to Figure 1).**

**Figure S3. Divergent fungal richness maturation patterns are observed in the first year of life (related to Figure 2).**

**Figure S4. Individual-level taxonomic differences between infants with an increasing vs. decreasing bacterial alpha diversity trend at 3 and 12 months (related to Figure 3 and Table S2).**

**Figure S5. Individual-level taxonomic differences between infants with a decreasing vs. increasing fungal alpha diversity trend at 3 and 12 months (related to Figure 3 and Table S3).**

**Figure S6. Differences in bacterial co-occurrence networks are observed between increasing and decreasing alpha diversity trends at 3 and 12 months (related to Figure 4 and Table S5).**

**Figure S7. Differences in fungal co-occurrence networks are observed between increasing and decreasing alpha diversity trends at 3 and 12 months (related to Figure 4 and Table S6).**

**Figure S8. Differences in inter-kingdom co-occurrence networks are observed between infants with a typical (inverse) bacterial and fungal alpha diversity trend and atypical changes in bacterial, fungal, or both alpha diversity trends at 3 and 12 months (related to Figure 4 and Table S7).**

**Table S1. 16S and ITS2 read counts before and after sequence processing with the *DADA2* pipeline (related to Figure S1).**

|  | **16S** | | | **ITS2** | | |
| --- | --- | --- | --- | --- | --- | --- |
| *Pre-DADA2 Processing* | | | | | | |
|  | Median | Q1 | Q3 | Median | Q1 | Q3 |
| Overall | 40,827 | 35,938 | 46,699 | 22,715 | 13,613 | 45,114 |
| 3 Months | 41,460 | 37,562 | 48,231 | 20,116 | 11,073 | 37,050 |
| 12 Months | 39,790 | 33,785 | 45,865 | 26,259 | 16,566 | 45,803 |
| Positive | 38,557 | 35,161 | 47,503 | 167,376 | 102,699 | 222,283 |
| Negative | 407 | 262 | 1,101 | 2,247 | 2,175 | 7,618 |
| *Post-DADA2 Processing* | | | | | | |
|  | Median | Q1 | Q3 | Median | Q1 | Q3 |
| Overall | 33,103 | 27,700 | 37,369 | 15,740 | 9,625 | 29,954 |
| 3 Months | 34,818 | 29,337 | 39,159 | 13,918 | 7,597 | 23,642 |
| 12 Months | 30,545 | 25,707 | 35,401 | 19,734 | 12,054 | 35,110 |
| Positive | 36,053 | 33,758 | 38,562 | 162,985 | 45,353 | 216,782 |
| Negative | 62 | 51 | 94 | 386 | 137 | 464 |

Data presented as median, first quartile (Q1) or 25^th^ percentile, and third quartile (Q3) or 75^th^ percentile. Overall represents both 3- and 12-month samples from the CHILD Study.

**Table S2. Differences in CLR-transformed abundance of the top 15 bacterial genera by infant age and bacterial alpha diversity trend (related to Figure 3A-B and Figure S4).**

| **Genus** | **Infant Age** | **Alpha Diversity Trend (3 Months)** | **Alpha Diversity Trend (12 Months)** |
| --- | --- | --- | --- |
| *Akkermansia* | 0.843 | 0.695 | 0.118 |
| *Bacteroides* | **0.019** | 0.393 | **0.016** |
| *Bifidobacterium* | **<0.001** | 0.397 | 0.312 |
| *Blautia* | **<0.001** | 0.517 | 0.304 |
| *Enterobacteriaceae* Uncl. | **<0.001** | 0.924 | 0.527 |
| *Escherichia* | **<0.001** | **0.013** | 0.095 |
| *Faecalibacterium* | **<0.001** | 0.134 | 0.544 |
| *Haemophilus* | 0.248 | 0.506 | 0.724 |
| *Klebsiella* | **<0.001** | 0.123 | 0.064 |
| *Lachnospiraceae* Uncl. | **<0.001** | 0.641 | 0.208 |
| *Parabacteroides* | 0.348 | 0.460 | 0.281 |
| *Rikenellaceae* Uncl. | 0.539 | 0.665 | 0.190 |
| *Roseburia* | **<0.001** | 0.149 | 0.324 |
| *Ruminococcus* | 0.135 | 0.695 | 0.620 |
| *Veillonella* | **<0.001** | 0.236 | 0.588 |

Top 15 bacterial genera assessed for normality using the Shapiro-Wilk test. Non-normally distributed CLR-transformed abundances were assessed by Mann-Whitney U test. Normally distributed CLR-transformed abundances were assessed for equality of variance using the F-test, then evaluated by Student’s t-test or Welch’s Two Sample t-test if the variance was equal or unequal, respectively. Data are reported as p-values. Significant p-values (<0.05) are bolded.

Centered log-ratio, CLR; unclassified, Uncl.

**Table S3. Differences in CLR-transformed abundance of the top 15 fungal genera by infant age and fungal alpha diversity trend (related to Figure 3D-E and Figure S5).**

| **Genus** | **Infant Age** | **Alpha Diversity Trend (3 Months)** | **Alpha Diversity Trend (12 Months)** |
| --- | --- | --- | --- |
| *Alternaria* | 0.864 | 0.336 | **0.001** |
| *Candida* | 0.314 | **<0.001** | **0.001** |
| *Cladosporium* | **<0.001** | **0.038** | 0.784 |
| *Debaryomyces* | **<0.001** | 0.139 | 0.068 |
| *Fomitopsis* | **<0.001** | 0.258 | 0.076 |
| *Ganoderma* | 0.067 | 0.893 | 0.854 |
| *Malassezia* | **<0.001** | **0.039** | 0.112 |
| *Meyerozyma* | **<0.001** | **<0.001** | 0.069 |
| *Mycosphaerella* | **<0.001** | 0.069 | 0.394 |
| *Naganishia* | **<0.001** | **0.006** | 0.400 |
| *Resinicium* | 0.098 | 0.126 | 0.254 |
| *Rhodotorula* | **0.013** | 0.854 | 0.147 |
| *Rigidoporus* | 0.458 | 0.917 | **0.033** |
| *Saccharomyces* | **<0.001** | 0.190 | **0.005** |
| *Sclerotiniaceae* Uncl. | **0.002** | **0.014** | 0.068 |

Top 15 fungal genera assessed for normality using the Shapiro-Wilk test. Non-normally distributed CLR-transformed abundances were assessed by Mann-Whitney U test. Normally distributed CLR-transformed abundances were assessed for equality of variance using the F-test, then evaluated by Student’s t-test of Welch’s Two Sample t-test if the variance was equal or unequal, respectively. Data are reported as p-values. Significant p-values (<0.05) are bolded.

Centered log-ratio, CLR; unclassified, Uncl.

**Table S4. Pair-wise comparison of typical (inverse), bacteria atypical, and fungi atypical inter-kingdom microbial co-occurrence network properties at 3 and 12 months (related to Figure 4).**

| **3 Months – Typical (Inverse) vs. Bacteria Atypical** | | | | | | | | | |
| --- | --- | --- | --- | --- | --- | --- | --- | --- | --- |
|  | **Typical (Inverse)** | | | | **Bacteria Atypical** | | **Absolute Difference** | | **p-value** |
| **Global Network Measures** | | | | | | | | | |
| Clustering Coefficient | 0.262 | | | | 0.236 | | 0.027 | | 0.847 |
| Modularity | 0.552 | | | | 0.359 | | 0.192 | | 0.355 |
| Positive Edge Percentage | 93.750 | | | | 56.997 | | 36.753 | | 0.079 |
| Edge Density | 0.098 | | | | 0.080 | | 0.018 | | 0.744 |
| Natural Connectivity | 0.059 | | | | 0.020 | | 0.039 | | 0.653 |
| Vertex Connectivity | 1.000 | | | | 1.000 | | 0.000 | | 1.000 |
| Edge Connectivity | 1.000 | | | | 1.000 | | 0.000 | | 1.000 |
| Average Dissimilarity | 0.952 | | | | 0.972 | | 0.020 | | 0.634 |
| Average Path Length | 2.536 | | | | 1.607 | | 0.928 | | 0.249 |
| **Jaccard Index Values** | | | | | | | | | |
|  | | | **Jacc** | | | **P<=Jacc** | | **P>=Jacc** | |
| Degree | | | 0.222 | | | 0.105 | | 0.949 | |
| Betweenness Centrality | | | 0.147 | | | **0.013** | | 0.996 | |
| Closeness Centrality | | | 0.222 | | | 0.105 | | 0.949 | |
| Eigenvector Centrality | | | 0.128 | | | **0.003** | | 0.999 | |
| Hub Taxa | | | 0.000 | | | **0.026** | | 1.000 | |
| **3 Months – Typical (Inverse) vs. Fungi Atypical** | | | | | | | | | |
|  | **Typical (Inverse)** | | | | **Fungi Atypical** | | **Absolute Difference** | | **p-value** |
| **Global Network Measures** | | | | | | | | | |
| Clustering Coefficient | 0.262 | | | | 0.394 | | 0.132 | | 0.463 |
| Modularity | 0.552 | | | | 0.265 | | 0.287 | | 0.394 |
| Positive Edge Percentage | 93.750 | | | | 56.579 | | 37.171 | | 0.158 |
| Edge Density | 0.098 | | | | 0.146 | | 0.047 | | 0.445 |
| Natural Connectivity | 0.059 | | | | 0.056 | | 0.003 | | 0.922 |
| Vertex Connectivity | 1.000 | | | | 3.000 | | 2.000 | | 0.676 |
| Edge Connectivity | 1.000 | | | | 3.000 | | 2.000 | | 0.683 |
| Average Dissimilarity | 0.952 | | | | 0.945 | | 0.008 | | 0.761 |
| Average Path Length | 2.536 | | | | 1.316 | | 1.220 | | 0.338 |
| **Jaccard Index Values** | | | | | | | | | |
|  | | | **Jacc** | | | **P<=Jacc** | | **P>=Jacc** | |
| Degree | | | 0.128 | | | **0.003** | | 0.999 | |
| Betweenness Centrality | | | 0.147 | | | **0.013** | | 0.996 | |
| Closeness Centrality | | | 0.189 | | | **0.041** | | 0.983 | |
| Eigenvector Centrality | | | 0.158 | | | **0.013** | | 0.996 | |
| Hub Taxa | | | 0.125 | | | 0.195 | | 0.961 | |
| **3 Months – Bacteria Atypical vs. Fungi Atypical** | | | | | | | | | |
|  | | **Bacteria Atypical** | | **Fungi Atypical** | | | **Absolute Difference** | | **p-value** |
| **Global Network Measures** | | | | | | | | | |
| Clustering Coefficient | | 0.236 | | 0.394 | | | 0.158 | | **0.022** |
| Modularity | | 0.359 | | 0.265 | | | 0.095 | | 0.462 |
| Positive Edge Percentage | | 56.997 | | 56.579 | | | 0.418 | | 0.971 |
| Edge Density | | 0.080 | | 0.146 | | | 0.065 | | 0.073 |
| Natural Connectivity | | 0.020 | | 0.056 | | | 0.036 | | **0.017** |
| Vertex Connectivity | | 1.000 | | 3.000 | | | 2.000 | | 0.646 |
| Edge Connectivity | | 1.000 | | 3.000 | | | 2.000 | | 0.646 |
| Average Dissimilarity | | 0.972 | | 0.945 | | | 0.027 | | **0.011** |
| Average Path Length | | 1.607 | | 1.316 | | | 0.291 | | 0.315 |
| **Jaccard Index Values** | | | | | | | | | |
|  | | | **Jacc** | | | **P<=Jacc** | | **P>=Jacc** | |
| Degree | | | 0.100 | | | **<0.001** | | 0.999 | |
| Betweenness Centrality | | | 0.128 | | | **0.003** | | 0.999 | |
| Closeness Centrality | | | 0.128 | | | **0.003** | | 0.999 | |
| Eigenvector Centrality | | | 0.158 | | | **0.013** | | 0.996 | |
| Hub Taxa | | | 0.250 | | | 0.468 | | 0.805 | |
| **12 Months – Typical (Inverse) vs. Bacteria Atypical** | | | | | | | | | |
|  | | **Typical (Inverse)** | | **Bacteria Atypical** | | | **Absolute Difference** | | **p-value** |
| **Global Network Measures** | | | | | | | | | |
| Clustering Coefficient | | 0.277 | | 0.317 | | | 0.040 | | 0.585 |
| Modularity | | 0.597 | | 0.280 | | | 0.317 | | 0.380 |
| Positive Edge Percentage | | 70.370 | | 57.104 | | | 13.267 | | 0.712 |
| Edge Density | | 0.054 | | 0.091 | | | 0.038 | | 0.658 |
| Natural Connectivity | | 0.023 | | 0.022 | | | 0.001 | | 0.798 |
| Vertex Connectivity | | 1.000 | | 3.000 | | | 2.000 | | 1.000 |
| Edge Connectivity | | 1.000 | | 3.000 | | | 2.000 | | 1.000 |
| Average Dissimilarity | | 0.979 | | 0.968 | | | 0.011 | | 0.563 |
| Average Path Length | | 2.810 | | 1.577 | | | 1.233 | | 0.669 |
| **Jaccard Index Values** | | | | | | | | | |
|  | | | **Jacc** | | | **P<=Jacc** | | **P>=Jacc** | |
| Degree | | | 0.122 | | | **0.002** | | 0.999 | |
| Betweenness Centrality | | | 0.179 | | | **0.027** | | 0.990 | |
| Closeness Centrality | | | 0.211 | | | 0.072 | | 0.968 | |
| Eigenvector Centrality | | | 0.211 | | | 0.072 | | 0.968 | |
| Hub Taxa | | | 0.000 | | | **0.017** | | 1.00 | |
| **12 Months – Typical (Inverse) vs. Fungi Atypical** | | | | | | | | | |
|  | | **Typical (Inverse)** | | **Fungi Atypical** | | | **Absolute Difference** | | **p-value** |
| **Global Network Measures** | | | | | | | | | |
| Clustering Coefficient | | 0.277 | | 0.316 | | | 0.038 | | 0.776 |
| Modularity | | 0.597 | | 0.190 | | | 0.407 | | 0.385 |
| Positive Edge Percentage | | 70.370 | | 51.876 | | | 18.494 | | 0.628 |
| Edge Density | | 0.054 | | 0.153 | | | 0.099 | | 0.396 |
| Natural Connectivity | | 0.023 | | 0.030 | | | 0.007 | | 0.548 |
| Vertex Connectivity | | 1.000 | | 4.000 | | | 3.000 | | 0.558 |
| Edge Connectivity | | 1.000 | | 4.000 | | | 3.000 | | 0.561 |
| Average Dissimilarity | | 0.979 | | 0.949 | | | 0.030 | | 0.450 |
| Average Path Length | | 2.810 | | 1.260 | | | 1.550 | | 0.572 |
| **Jaccard Index Values** | | | | | | | | | |
|  | | | **Jacc** | | | **P<=Jacc** | | **P>=Jacc** | |
| Degree | | | 0.179 | | | **0.027** | | 0.990 | |
| Betweenness Centrality | | | 0.211 | | | 0.072 | | 0.968 | |
| Closeness Centrality | | | 0.211 | | | 0.072 | | 0.968 | |
| Eigenvector Centrality | | | 0.150 | | | **0.008** | | 0.997 | |
| Hub Taxa | | | 0.000 | | | **0.017** | | 1.000 | |
| **12 Months – Bacteria Atypical vs. Fungi Atypical** | | | | | | | | | |
|  | | **Bacteria Atypical** | | **Fungi Atypical** | | | **Absolute Difference** | | **p-value** |
| **Global Network Measures** | | | | | | | | | |
| Clustering Coefficient | | 0.317 | | 0.316 | | | 0.001 | | 0.989 |
| Modularity | | 0.280 | | 0.190 | | | 0.090 | | 0.421 |
| Positive Edge Percentage | | 57.104 | | 51.876 | | | 5.228 | | 0.365 |
| Edge Density | | 0.091 | | 0.153 | | | 0.062 | | 0.193 |
| Natural Connectivity | | 0.022 | | 0.030 | | | 0.008 | | 0.532 |
| Vertex Connectivity | | 1.000 | | 4.000 | | | 3.000 | | 0.353 |
| Edge Connectivity | | 1.000 | | 4.000 | | | 3.000 | | 0.357 |
| Average Dissimilarity | | 0.968 | | 0.949 | | | 0.018 | | 0.364 |
| Average Path Length | | 1.577 | | 1.260 | | | 0.317 | | 0.107 |
| **Jaccard Index Values** | | | | | | | | | |
|  | | | **Jacc** | | | **P<=Jacc** | | **P>=Jacc** | |
| Degree | | | 0.070 | | | **<0.001** | | 0.999 | |
| Betweenness Centrality | | | 0.211 | | | 0.072 | | 0.967 | |
| Closeness Centrality | | | 0.179 | | | **0.026** | | 0.990 | |
| Eigenvector Centrality | | | 0.095 | | | **<0.001** | | 0.999 | |
| Hub Taxa | | | 0.111 | | | 0.143 | | 0.974 | |

**Table S5. Bacterial co-occurrence network properties between typical and atypical alpha diversity trends at 3 and 12 months (related to Figure S6).**

| **3 Months** | | | | | | | |
| --- | --- | --- | --- | --- | --- | --- | --- |
|  | **Increase (Typical)** | | **Decrease (Atypical)** | | **Absolute Difference** | | **p-value** |
| **Global Network Measures** | | | | | | | |
| Clustering Coefficient | 0.561 | | 0.273 | | 0.880 | | 0.558 |
| Modularity | 0.080 | | 0.427 | | 0.288 | | 0.344 |
| Positive Edge Percentage | 100.000 | | 62.832 | | 37.168 | | 0.254 |
| Edge Density | 0.500 | | 0.096 | | 0.404 | | 0.411 |
| Vertex Connectivity | 1.000 | | 1.000 | | 0.000 | | 1.000 |
| Edge Connectivity | 1.000 | | 1.000 | | 0.000 | | 1.000 |
| Average Path Length | 1.050 | | 1.871 | | 0.822 | | 0.260 |
| **Jaccard Index Values** | | | | | | | |
|  | | **Jacc** | | **P<=Jacc** | | **P>=Jacc** | |
| Degree | | 0.200 | | 0.209 | | 0.921 | |
| Betweenness Centrality | | 0.000 | | **0.002** | | 1.000 | |
| Closeness Centrality | | 0.200 | | 0.209 | | 0.921 | |
| Eigenvector Centrality | | 0.059 | | **0.009** | | 0.999 | |
| Hub Taxa | | 0.000 | | 0.132 | | 1.000 | |
| **12 Months** | | | | | | | |
|  | **Increase (Typical)** | | **Decrease (Atypical)** | | **Absolute Difference** | | **p-value** |
| **Global Network Measures** | | | | | | | |
| Clustering Coefficient | 0.161 | | 0.272 | | 0.111 | | 0.504 |
| Modularity | 0.403 | | 0.357 | | 0.046 | | 0.686 |
| Positive Edge Percentage | 72.222 | | 59.615 | | 12.607 | | 0.424 |
| Edge Density | 0.171 | | 0.091 | | 0.080 | | 0.686 |
| Vertex Connectivity | 1.000 | | 1.000 | | 0.000 | | 1.000 |
| Edge Connectivity | 1.000 | | 1.000 | | 0.000 | | 1.000 |
| Average Path Length | 2.085 | | 1.740 | | 0.344 | | 0.289 |
| **Jaccard Index Values** | | | | | | | |
|  | | **Jacc** | | **P<=Jacc** | | **P>=Jacc** | |
| Degree | | 0.111 | | **0.008** | | 0.998 | |
| Betweenness centrality | | 0.150 | | 0.060 | | 0.982 | |
| Closeness centrality | | 0.200 | | 0.112 | | 0.954 | |
| Eigenvector Centrality | | 0.250 | | 0.263 | | 0.862 | |
| Hub Taxa | | 0.500 | | 0.889 | | 0.407 | |

**Table S6. Fungal co-occurrence network properties between typical and atypical alpha diversity trends at 3 and 12 months (related to Figure S7).**

| **3 Months** | | | | | | | |
| --- | --- | --- | --- | --- | --- | --- | --- |
|  | **Decrease (Typical)** | | **Increase (Atypical)** | | **Absolute Difference** | | **p-value** |
| **Global Network Measures** | | | | | | | |
| Clustering Coefficient | 0.000 | | 0.353 | | 0.353 | | 0.841 |
| Modularity | 0.167 | | 0.434 | | 0.268 | | 0.926 |
| Positive Edge Percentage | 100.000 | | 59.459 | | 40.541 | | 0.423 |
| Edge Density | 0.500 | | 0.124 | | 0.376 | | 0.958 |
| Vertex Connectivity | 1.000 | | 1.000 | | 0.000 | | 1.000 |
| Edge Connectivity | 1.000 | | 1.000 | | 0.000 | | 1.000 |
| Average Path Length | 1.146 | | 1.770 | | 0.624 | | 0.947 |
| **Jaccard Index Values** | | | | | | | |
|  | | **Jacc** | | **P<=Jacc** | | **P>=Jacc** | |
| Degree | | 0.300 | | 0.559 | | 0.701 | |
| Betweenness Centrality | | 0.000 | | **0.012** | | 1.000 | |
| Closeness Centrality | | 0.083 | | 0.054 | | 0.992 | |
| Eigenvector Centrality | | 0.444 | | 0.855 | | 0.350 | |
| Hub Taxa | | 0.000 | | 0.198 | | 1.000 | |
| **12 Months** | | | | | | | |
|  | **Decrease (Typical)** | | **Increase (Atypical)** | | **Absolute Difference** | | **p-value** |
| **Global Network Measures** | | | | | | | |
| Clustering Coefficient | 0.257 | | 0.343 | | 0.087 | | 0.710 |
| Modularity | 0.272 | | 0.356 | | 0.084 | | 0.750 |
| Positive Edge Percentage | 100.000 | | 51.020 | | 48.980 | | 0.112 |
| Edge Density | 0.321 | | 0.163 | | 0.158 | | 0.725 |
| Vertex Connectivity | 1.000 | | 1.000 | | 0.000 | | 1.000 |
| Edge Connectivity | 1.000 | | 1.000 | | 0.000 | | 1.000 |
| Average Path Length | 1.293 | | 1.776 | | 0.483 | | 0.525 |
| **Jaccard Index Values** | | | | | | | |
|  | | **Jacc** | | **P<=Jacc** | | **P>=Jacc** | |
| Degree | | 0.167 | | 0.181 | | 0.946 | |
| Betweenness centrality | | 0.222 | | 0.377 | | 0.857 | |
| Closeness centrality | | 0.273 | | 0.473 | | 0.766 | |
| Eigenvector Centrality | | 0.167 | | 0.181 | | 0.946 | |
| Hub Taxa | | 0.000 | | 0.198 | | 1.000 | |

**Table S7. Inter-kingdom co-occurrence network properties between typical and atypical (bacteria, fungi, or both) alpha diversity trends at 3 and 12 months (related to Figure S8).**

| **3 Months** | | | | | | | |
| --- | --- | --- | --- | --- | --- | --- | --- |
|  | **Typical (Inverse)** | | **Atypical (Bacteria, Fungi or Both)** | | **Absolute Difference** | | **p-value** |
| **Global Network Measures** | | | | | | | |
| Clustering Coefficient | 0.262 | | 0.849 | | 0.122 | | 0.498 |
| Modularity | 0.552 | | 0.140 | | 0.139 | | 0.439 |
| Positive Edge Percentage | 93.750 | | 78.409 | | 15.341 | | 0.197 |
| Edge Density | 0.098 | | 0.033 | | 0.065 | | 0.560 |
| Natural Connectivity | 0.059 | | 0.018 | | 0.041 | | 0.577 |
| Vertex Connectivity | 1.000 | | 1.000 | | 0.000 | | 1.000 |
| Edge Connectivity | 1.000 | | 1.000 | | 0.000 | | 1.000 |
| Average Dissimilarity | 0.952 | | 0.986 | | 0.034 | | 0.519 |
| Average Path Length | 2.536 | | 3.565 | | 1.029 | | 0.389 |
| **Jaccard Index Values** | | | | | | | |
|  | | **Jacc** | | **P<=Jacc** | | **P>=Jacc** | |
| Degree | | 0.419 | | 0.885 | | 0.203 | |
| Betweenness Centrality | | 0.300 | | 0.432 | | 0.714 | |
| Closeness Centrality | | 0.257 | | 0.221 | | 0.873 | |
| Eigenvector Centrality | | 0.257 | | 0.221 | | 0.873 | |
| Hub Taxa | | 0.125 | | 0.195 | | 0.961 | |
| **12 Months** | | | | | | | |
|  | **Typical (Inverse)** | | **Atypical (Bacteria, Fungi or Both)** | | **Absolute Difference** | | **p-value** |
| **Global Network Measures** | | | | | | | |
| Clustering Coefficient | 0.277 | | 0.310 | | 0.033 | | 0.710 |
| Modularity | 0.597 | | 0.533 | | 0.065 | | 0.493 |
| Positive Edge Percentage | 70.370 | | 64.912 | | 5.458 | | 0.589 |
| Edge Density | 0.054 | | 0.052 | | 0.002 | | 0.850 |
| Natural Connectivity | 0.023 | | 0.021 | | 0.002 | | 0.888 |
| Vertex Connectivity | 1.000 | | 1.000 | | 0.000 | | 1.000 |
| Edge Connectivity | 1.000 | | 1.000 | | 0.000 | | 1.000 |
| Average Dissimilarity | 0.979 | | 0.981 | | 0.002 | | 0.769 |
| Average Path Length | 2.810 | | 2.577 | | 0.233 | | 0.717 |
| **Jaccard Index Values** | | | | | | | |
|  | | **Jacc** | | **P<=Jacc** | | **P>=Jacc** | |
| Degree | | 0.278 | | 0.303 | | 0.810 | |
| Betweenness centrality | | 0.150 | | **0.008** | | 0.997 | |
| Closeness centrality | | 0.278 | | 0.303 | | 0.810 | |
| Eigenvector Centrality | | 0.243 | | 0.162 | | 0.913 | |
| Hub Taxa | | 0.000 | | **0.017** | | 1.000 | |

**Table S8. Logistic regression statistics between maternal, infant, and early-life factors and bacterial alpha diversity trend (related to Figure 4B).**

Odds ratio, OR; confidence interval, CI

| **Characteristics** | **OR** | **95% CI** | **z-value** | **Pr (>\|z\|)** |
| --- | --- | --- | --- | --- |
| Infant Sex (Female) | 0.45 | 0.10-1.79 | -1.09 | 0.276 |
| Birth Mode (C-Section) | 11.57 | 1.76-112.13 | 2.35 | **0.019** |
| Partial Breastfeeding (3 months) | 0.16 | 0.03-0.75 | -2.18 | **0.029** |
| Exclusive Breastfeeding (3 months) | 0.05 | 0.00-0.29 | -2.98 | **0.003** |
| Age at Introduction of Solid Foods (months) | 0.90 | 0.54-1.51 | -0.41 | 0.684 |
| Infant *FUT2* Genotype – AG | 3.76 | 0.62-33.92 | 1.35 | 0.178 |
| Infant *FUT2* Genotype – GG | 23.02 | 2.96-280.06 | 2.76 | **0.006** |
| Maternal *FUT2* Genotype – AG | 0.29 | 0.04-1.79 | -1.33 | 0.183 |
| Maternal *FUT2* Genotype – GG | 0.04 | 0.00-0.43 | -2.57 | **0.010** |
| Prenatal Antibiotics | 15.80 | 1.96-194.97 | 2.40 | **0.017** |
| Intrapartum Antibiotics | 0.27 | 0.04-1.59 | -1.39 | 0.163 |
| Maternal Healthy Eating Index  (per 10 units) | 0.89 | 0.36-2.28 | -0.26 | 0.796 |
| Maternal Artificially Sweetened Beverage Consumption in Pregnancy | 0.39 | 0.10-1.42 | -1.39 | 0.165 |

**Table S9. Logistic regression statistics between maternal, infant, and early-life factors and fungal alpha diversity trend (related to Figure 4D).**

Odds ratio, OR; confidence interval, CI

| **Characteristics** | **OR** | **95% CI** | **z-value** | **Pr (>\|z\|)** |
| --- | --- | --- | --- | --- |
| Infant Sex (Female) | 2.54 | 0.70-10.21 | 1.38 | 0.167 |
| Birth Mode (C-Section) | 0.66 | 0.12-3.43 | -0.49 | 0.626 |
| Partial Breastfeeding (3 months) | 2.07 | 0.37-12.74 | 0.83 | 0.408 |
| Exclusive Breastfeeding (3 months) | 0.86 | 0.13-5.40 | -0.16 | 0.875 |
| Age at Introduction of Solid Foods (months) | 1.22 | 0.63-2.52 | 0.56 | 0.574 |
| Infant *FUT2* Genotype – AG | 3.91 | 0.57-39.72 | 1.29 | 0.196 |
| Infant *FUT2* Genotype – GG | 4.05 | 0.44-52.15 | 1.17 | 0.240 |
| Maternal *FUT2* Genotype – AG | 0.80 | 0.13-5.04 | -0.24 | 0.809 |
| Maternal *FUT2* Genotype – GG | 0.51 | 0.05-4.38 | -0.61 | 0.541 |
| Intrapartum Antibiotics | 0.35 | 0.07-1.48 | -1.38 | 0.169 |
| Infant Antibiotic Exposure (3-12 months) | 1.48 | 0.27-7.89 | 0.47 | 0.642 |
| Maternal Healthy Eating Index  (per 10 units) | 0.93 | 0.39-2.35 | -0.17 | 0.869 |
| Maternal Artificially Sweetened Beverage Consumption in Pregnancy | 8.32 | 1.98-48.59 | 2.66 | **0.008** |

**Figure S1. 16S and ITS2 sequencing depth and sample composition (related to Table S1).** A) Histogram of total reads per sample for 16S (bacterial) data after sequence processing with *DADA2*. B) Histogram of total reads per sample for ITS2 (fungal) data after sequence processing with *DADA2*. C) Comparison of sample vs. positive control composition for 16S (bacterial) data by principle coordinate analysis using the Bray-Curtis dissimilarity index. D) Comparison of sample vs. positive control composition for ITS2 (fungal) data by principle coordinate analysis using the Bray-Curtis dissimilarity index. Positive controls included both mock communities and single organisms.

**Figure S2. Divergent bacterial richness maturation patterns are observed in the first year of life (related to Figure 1).** A) Changes in bacterial richness (Chao1) per individual from 3 to 12 months, assessed by paired t-test (increase: n = 87, decrease: n = 11). B) Comparison of bacterial beta diversity by bacterial richness (Chao1) trend at 3 and 12 months, assessed by PERMANOVA (increase: n = 87, decrease: n = 11). Ellipses represent 95% CI. C) Relative abundance of the 15 most abundant bacterial genera by bacterial richness trend at 3 and 12 months (increase: n = 87, decrease: n = 11).

**Figure S3. Divergent fungal richness maturation patterns are observed in the first year of life (related to Figure 2).** A) Changes in fungal richness (Chao1) per individual from 3 to 12 months, assessed by paired t-test (decrease: n = 63, increase: n = 25, unchanged: n = 3). B) Comparison of fungal beta diversity by fungal richness (Chao1) trend at 3 and 12 months, assessed by PERMANOVA (decrease: n = 63, increase: n = 25, unchanged: n = 3). C) Relative abundance of the 15 most abundant fungal genera by fungal richness trend at 3 and 12 months (decrease: n = 63, increase: n = 25, unchanged: n = 3).

**
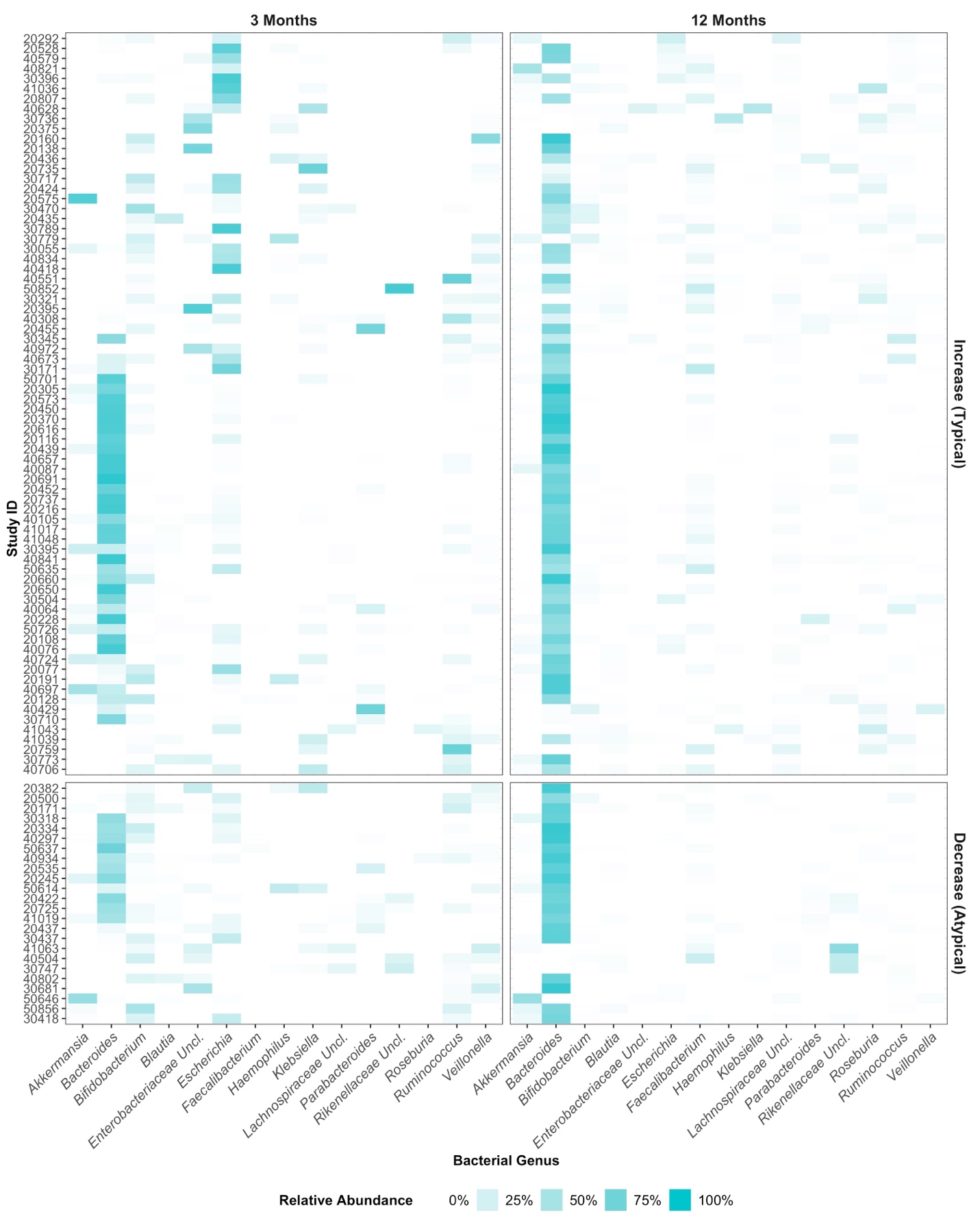
Figure S4. Individual-level taxonomic differences between infants with an increasing vs. decreasing bacterial alpha diversity trend at 3 and 12 months (related to Figure 3 and Table S2).** Relative abundance of the 15 most abundant bacterial genera per individual by bacterial alpha diversity trend at 3 and 12 months of age (increase: n = 74, decrease: n = 24).

**
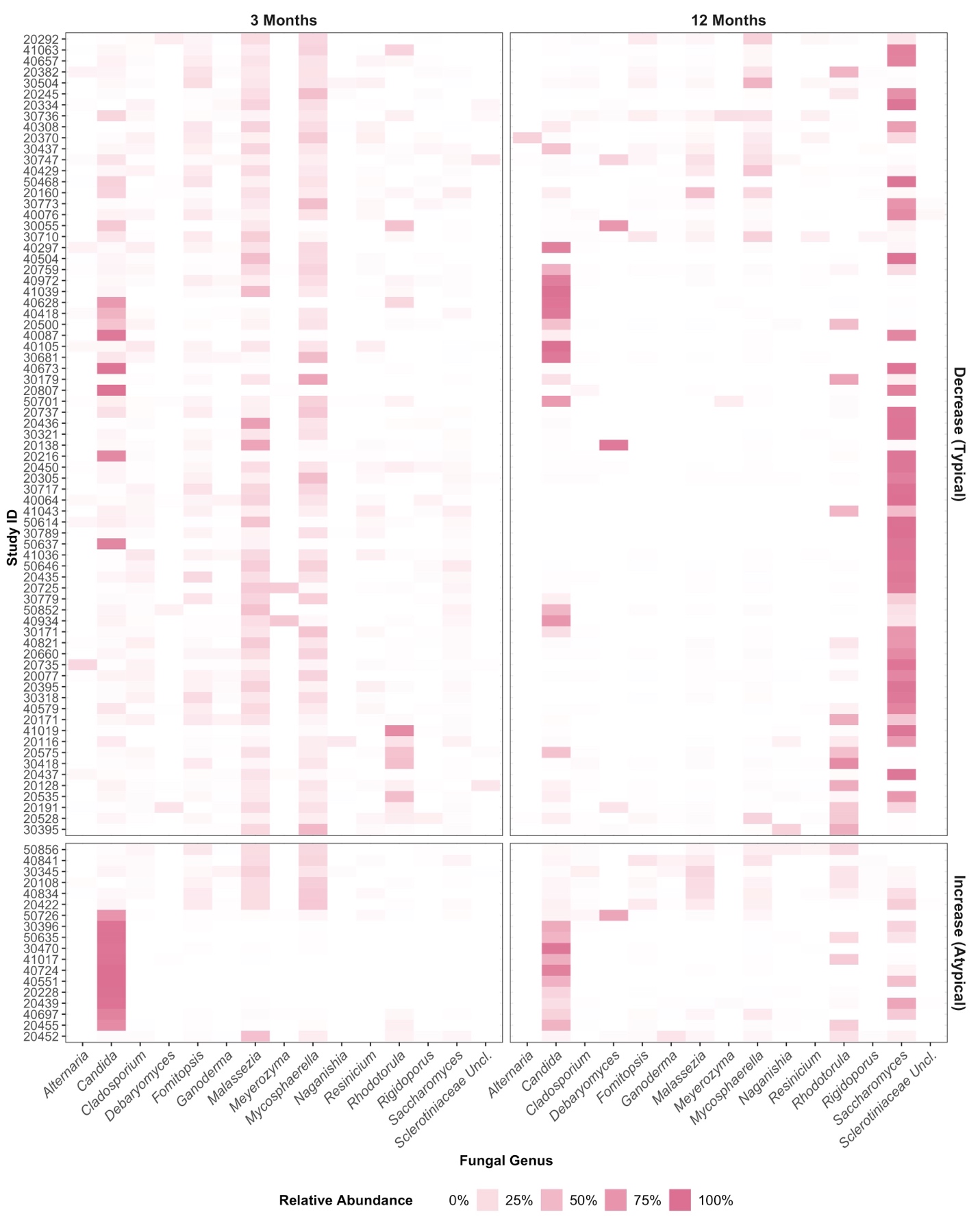
Figure S5. Individual-level taxonomic differences between infants with a decreasing vs. increasing fungal alpha diversity trend at 3 and 12 months (related to Figure 3 and Table S3).** Relative abundance of the 15 most abundant fungal genera per individual by fungal alpha diversity trend at 3 and 12 months of age (decrease: n = 73, increase: n = 18).

**Figure S6. Differences in bacterial co-occurrence networks are observed between increasing and decreasing alpha diversity trends at 3 and 12 months (related to Figure 4 and Table S5).** Correlation networks of bacterial species based on alpha diversity trend at A) 3 and B) 12 months. Infants were classified into increasing (n = 74) and decreasing (n = 24) bacterial alpha diversity trends based on changes from 3 to 12 months. Networks were generated using the fast greedy clustering algorithm with a minimum Pearson correlation coefficient threshold of 0.4. Positive correlations are displayed in green and negative correlations in red. Bacterial species are represented by circles. Hub taxa are those with the highest betweenness centrality and are labelled with their shape perimeter bolded. Shape colour represents clusters of species more likely to co-occur with one another than with species from outside of these modules. Pair-wise comparisons of network measures were calculated using 5,000 permutations (see Table S5).

**Figure S7. Differences in fungal co-occurrence networks are observed between increasing and decreasing alpha diversity trends at 3 and 12 months (related to Figure 4 and Table S6).** Correlation networks of fungal species based on alpha diversity trend at A) 3 and B) 12 months. Infants were classified into decreasing (n = 73) and increasing (n = 18) fungal alpha diversity trends based on changes from 3 to 12 months. Networks were generated using the fast greedy clustering algorithm with a minimum Pearson correlation coefficient threshold of 0.4. Positive correlations are displayed in green and negative correlations in red. Fungal species are represented by triangles. Hub taxa are those with the highest betweenness centrality and are labelled with their shape perimeter bolded. Shape colour represents clusters of species more likely to co-occur with one another than with species from outside of these modules. Pair-wise comparisons of network measures were calculated using 5,000 permutations (see Table S6).

**Figure S8. Differences in inter-kingdom co-occurrence networks are observed between infants with a typical (inverse) bacterial and fungal alpha diversity trend and atypical changes in bacterial, fungal, or both alpha diversity trends at 3 and 12 months (related to Figure 4 and Table S7).** Correlation networks of bacterial and fungal species based on overall alpha diversity relationships at A) 3 and B) 12 months. Infants were classified into overall alpha diversity relationships based on the combination of alpha diversity trends they exhibited for bacteria and fungi. A typical inverse relationship was characterized by increasing bacterial and decreasing fungal alpha diversity (n = 50) and an atypical relationship was characterized by atypical shifts in bacterial (n = 21), fungal (n = 16), or both bacterial and fungal alpha diversity (n = 2) and combined into one category (total n = 39). Networks were generated using the fast greedy clustering algorithm with a minimum Pearson correlation coefficient threshold of 0.4. Positive correlations are displayed in green and negative correlations in red. Bacterial species are represented by circles and fungal species by triangles. Hub taxa are those with the highest betweenness centrality and are labelled with their shape perimeter bolded. Shape colour represents clusters of species more likely to co-occur with one another than with species from outside of these modules. Pair-wise comparisons of network measures were calculated using 5,000 permutations (see Table S7).
